# Evidence of neolithic cannibalism among farming communities at El Mirador cave, Sierra de Atapuerca, Spain

**DOI:** 10.1038/s41598-025-10266-w

**Published:** 2025-08-07

**Authors:** Palmira Saladié, Francesc Marginedas, Juan Ignacio Morales, Josep María Vergès, Ethel Allué, Isabel Expósito, Marina Lozano, Patricia Martín, Javier Iglesias-Bexiga, Marta Fontanals, Roser Marsal, Raquel Hernando, Aitor Burguet-Coca, Antonio Rodríguez-Hidalgo

**Affiliations:** 1 Catalan Institute of Human Paleoecology and Social Evolution (IPHES), Zona Educacional 4, Campus Sescelades URV (Building W3), Tarragona, 43007 Spain; 2 Area of Prehistory, Rovira i Virgili University, Campus Catalunya. Avinguda de Catalunya 35, Tarragona, 43003 Spain; 3 Department of Paleobiology, Unit Associated with CSIC, National Museum of Natural Sciences (CSIC), Calle José Gutiérrez Abascal, 2, Madrid, 28006 Spain; 4 Department of Biology, Autonomous University of Madrid (UAM), C/ Francisco Tomás y Valiente 1 Campus de Cantoblanco, Madrid, 28-049 Spain; 5 Institute of Near Eastern Ancient Studies (IEPOA), Autonomous University of Barcelona, Research Module A (MRA), UAB Campus, 08193, Bellaterra, Cerdanyola del Vallès, 08028 Spain; 6 National Center for Research on Human Evolution (CENIEH), Paseo Sierra de Atapuerca, 3, Burgos, 09002 Spain; 7 Faculty of Archaeology, Department of Archaeological Sciences, Leiden University, Einsteinweg 2, 2333CC, Leiden, The Netherlands; 8 Present Address: Institute of Archaeology-Mérida (CSIC-Junta de Extremadura), Spanish National Research Council, Plaza de España 15, Mérida, 06800 Spain

**Keywords:** Anthropology, Archaeology

## Abstract

In El Mirador cave in Sierra de Atapuerca, Spain, a unique collection of human remains provides insights into cannibalistic practices from the Neolithic to the Bronze Age. Six Early Bronze Age individuals (4600–4100 cal BP) showing signs of cannibalism were discovered in the early 2000s. Later excavations uncovered older remains with similar cultural modifications. A Bayesian statistical analysis of the radiocarbon dates identified a single earlier event (5709–5573 cal BP) unrelated to the Bronze Age finds. 87Sr/86Sr analysis showed the cannibalised people were of local origin. The episode coincided with the end of the Neolithic occupation, suggesting this was a not common behaviour among the cave inhabitants. Given the age of the cannibalised individuals and environmental conditions, the data does not indicate a response to famine. This study complements and expands upon our understanding of European prehistoric cannibalism. The current findings suggest that cannibalism may be linked to intergroup violence during late prehistoric periods.

**Supplementary Information:**

The online version contains supplementary material available at 10.1038/s41598-025-10266-w.

## Introduction

 Between the Neolithic and the Bell Beaker culture, the considerable diversity in funerary practices on the Iberian Peninsula poses challenges for interpreting how these societies approached death^1^. Early in this period (7500–6000 BP), evidence of corpse manipulation beyond the simple deposition of bodies is scarce^1,2^ but in later phases these kinds of funerary behaviours become more common in the archaeological record. These include secondary burials or depositions in which human bones are mixed with faunal remains, seeds, ceramics, and lithic artifacts; burials with grave goods alongside others lacking offerings^3–5^; and collective burials in megalithic structures and caves, which are common from the Middle Neolithic through the Chalcolithic. During the Bronze Age, funerary practices gradually shifted from collective to individual burials^6,7^.

While funerary rituals are widely documented, certain mortuary behaviours remain elusive. Early Neolithic human remains mixed with domestic items in habitational contexts are common^8^ but rarer practices related to the manipulation of dead bodies challenge straightforward interpretations of funerary intent^9,10^. These cases may reflect a broader interest in the body after death, one not necessarily associated with grief or commemoration^11^. Interpretations of such acts vary widely and include the symbolic internalisation of the deceased, the ritual destruction of an enemy’s body, or the treatment of remains with apparent emotional detachment^12–14^. These possibilities are not mutually exclusive and may, in fact, involve a range of emotional responses, from reverence to hostility, that flow between love and hate, depending on the cultural and relational context.

Cáceres and colleagues^15^ described a set of human remains from the Bronze Age level of MIR4 at El Mirador cave (Sierra de Atapuerca, Burgos, Spain), dated to 3,946 ± 385 cal BP. These remains exhibited abundant taphonomic modifications, which led the authors to interpret them as evidence of a cannibalism episode. Subsequent excavations in other areas of the cave have yielded additional human remains bearing cultural modifications. These new finds span different archaeological levels and are associated with varied uses of the site, including both livestock enclosures and burial contexts.

The aim of this article is to present and analyse the newly-discovered remains showing signs of anthropogenic modification, in order to expand the contribution to the understanding the forms and meanings of prehistoric cannibalism. Through detailed taphonomic analysis and direct radiocarbon dating of selected specimens, we seek to better understand the forms and meanings of cadaver manipulation at El Mirador. A key objective is to discern the context in which these processed remains originated, whether funerary, domestic, ritual, or otherwise. Additionally, ⁸⁷Sr/⁸⁶Sr isotope analysis was conducted to assess the geographic origins of the individuals involved. Finnaly, we expand the dataset available for the study of human cannibalism in the prehistory of Iberian Peninsula.

By integrating these results within the archaeological context, this study contributes to a broader discussion of mortuary and anthropophagic practices over time at the site. In particular, it allows us to explore potential cultural transformations and the factors, possibly diverse and overlapping, that may have influenced episodes of cannibalism. While the motivations behind these acts remain difficult to disentangle, this work aims to characterise the form(s) of cannibalism practiced at El Mirador, and to situate them within changing patterns of social behaviour among early farming communities.

## Archaeological context and human remains at El Mirador cave

El Mirador cave is part of a karst system located on the southern slope of the Sierra de Atapuerca (Supplementary note 1, Figure S1a). Archaeological work, which began in 1999 and is ongoing, has been carried out in three independent sectors: Test Pit (centre of the cave), Sector 100 (S100, north-western gallery), and Sector 200 (S200, north-eastern gallery) (Figures S1-S3). The layers of interest in this research are from different phases of occupation starting in the Neolithic and ending in the Middle Bronze Age, with associated^14^C dates ranging from 5,593 to 5,331 cal BP to 3,811–3,487 cal BP (Tables S1, S2). The site has yielded 5,056 human remains (Table S3) from four archaeological contexts. The study of the human remains from El Mirador Cave is authorized by the responsible of archaeological team and conducted in accordance with the international ethical guidelines on the study of ancient human remains.

First, a small pit containing 157 human remains and other unidentified bone fragments was excavated in Level MIR4 (Figure S4). Additionally, five human remains were found in MIR2 and MIR3, although the same origin was determined for the entire assemblage. This sample exhibited significant anthropogenic modifications (Table S3), which led to the proposal that they represented evidence of prehistoric human cannibalism^16^ dating to the beginning of the Bronze Age (Table S1). The presence of skull cups suggest the ceremonial consumption of these bodies, possibly involving the use of the cranial vaults as containers^17^. The Neolithic Test Pit levels (MIR24 to MIR6) are associated with livestock activities, and have been dated to 5593–5331 cal BP (Table S1). The Middle Bronze Age occupations are dated to 3811–3487 cal BP (MIR4 and MIR3A) (Tables S1, S2).

Secondly, an individual burial was also documented in an area excavated in Sector 100 (S100) (Figure S5). A nearly complete articulated skeleton of a young individual was recovered from Level MIR106 (Table S1). The body was placed on a rock shelf inside the cave^18^. Radiocarbon dating (3824–3575 cal BP) places the death and deposition in the Middle Bronze Age (Table S2, Table S3). No anthropogenic modifications were observed on these remains. Research on the different levels of Sector 100 indicates that the cave was primarily used as a livestock enclosure from the Neolithic to the Bronze Age, only interrupted by the individual inhumation^19^.

Thirdly, 4,097 human remains corresponding to a minimum of 38 individuals (MNI) were recovered from a small natural chamber in S200 used for collective funerary deposition during the Chalcolithic (Level MIR203), with ^14^C dating indicating a time span of 5214–4862 to 4786–4421 cal BP (Figure S6). The human remains were found accumulated and disarticulated^18^.

The fourth set of human remains was recovered during the excavation of S100 and S200. These bones were found mixed with material associated with the collective Chalcolithic burial and with activities related to the use of the cave as a sheepfold^20^. This assemblage is the specific focus of the present study.

## Results

Based on the taphonomic observations of the entire assemblage, this set of human remains was considered as a single group. It comprises 650 remains with anthropogenic modifications (Table S3), which were not observed in the remains from the funerary context but are present in those associated with the cannibalism event of MIR4. These traits led us to isolate this group of remains. Evidence of cremation, pot polishing, cut marks, percussion marks, peeling, and human tooth marks is present on different specimens in the sample (Table S4). We are aware that not all such modifications can be directly linked to intentional anthropogenic processing. Therefore, the set of specimens with signs directly related to the butchery processing of the skeletons (cut and percussion marks and peeling) consists of a total of 239 human remains (Table S4), each of which shows one or more of these modifications. The remains were recovered from different excavated levels, although most were found in reshuffled sediment layers and burrow fillings (Tables S3, S4). They were significant stratigraphically and spatially dispersed and mixed in with the remains of the collective burial and activities related to the sheepfold^23^.

### Dating

**Fig. 1:**
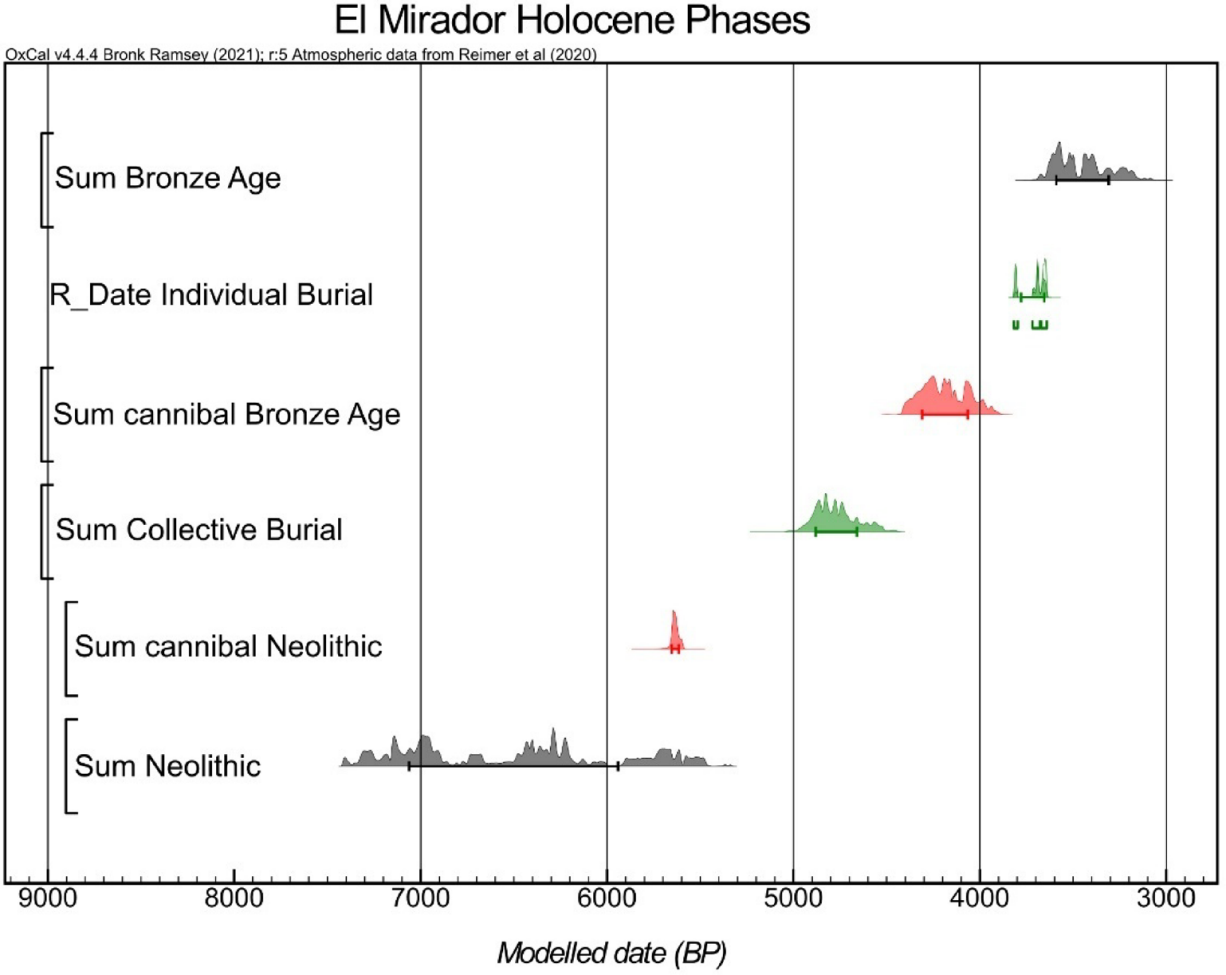
Bayesian chronological model of the Holocene sequence from El Mirador cave, generated using OxCal v4.4. The probability distributions represent the summed calibrated radiocarbon dates associated with each archaeological phase or event. Sum Bronze Age: all radiocarbon dates associated with Bronze Age contexts. R_Date Individual Burial: the calibrated date of the individual burial found in level MIR106. Sum Cannibal Bronze Age: dates associated with the cannibalised human remains from MIR4. Sum Collective Burial: dates from the Chalcolithic collective burial in level MIR203. Sum Cannibal Neolithic: newly analysed remains showing cultural modifications, presented in this study. Sum Neolithic: all radiocarbon dates from Neolithic levels.

Eight specimens from different archaeological levels with anthropogenic modifications (cut marks and percussion marks) were directly dated (Table S5, Figure S7). All the obtained dates exhibit remarkable consistency despite their dispersion across different units, including those composed of reshuffled levels and infilled burrows (Fig. 1, Table S2). The modeled chronology of the remains with anthropogenic modifications is 5709–5573 cal BP, which indicates that these remains correspond to slightly more recent occupations than those recorded in level MIR8^19^. No remains were recovered from occupations contemporaneous with the episode of cannibalism. The chronological homogeneity of the dated samples suggests that they result from a single occupational event, or various episodes separated by short time intervals. Thus, the specimens from S100 and S200 can be considered as a single assemblage.

The dispersion of cannibalised remains in El Mirador cave is linked to complex sedimentation processes, primarily anthropogenic in origin^21^. Throughout the Neolithic and most of the Middle and Late Bronze Age sequences the cave was repeatedly used for livestock activities^20,22^. The human remains found in S100 and levels MIR204 and MIR205 of S200 are associated with artifact dispersion from these activities, which led to the accumulation of manure and other archaeological remains, often subjected to periodic burning to sanitise the space and reduce dung volume^23,24^. Levels MIR101, MIR102, MIR201, MIR202, and MIR206 are reshuffled layers and infilled burrows. Manure in these contexts was frequently affected by bioturbation, as seen throughout the El Mirador cave sequence^24^. These factors suggest that the human-modified human bones were disturbed by sediment rearrangement, causing their dispersion and intermingling with other materials.

### MNI and age of death

**Fig. 2:**
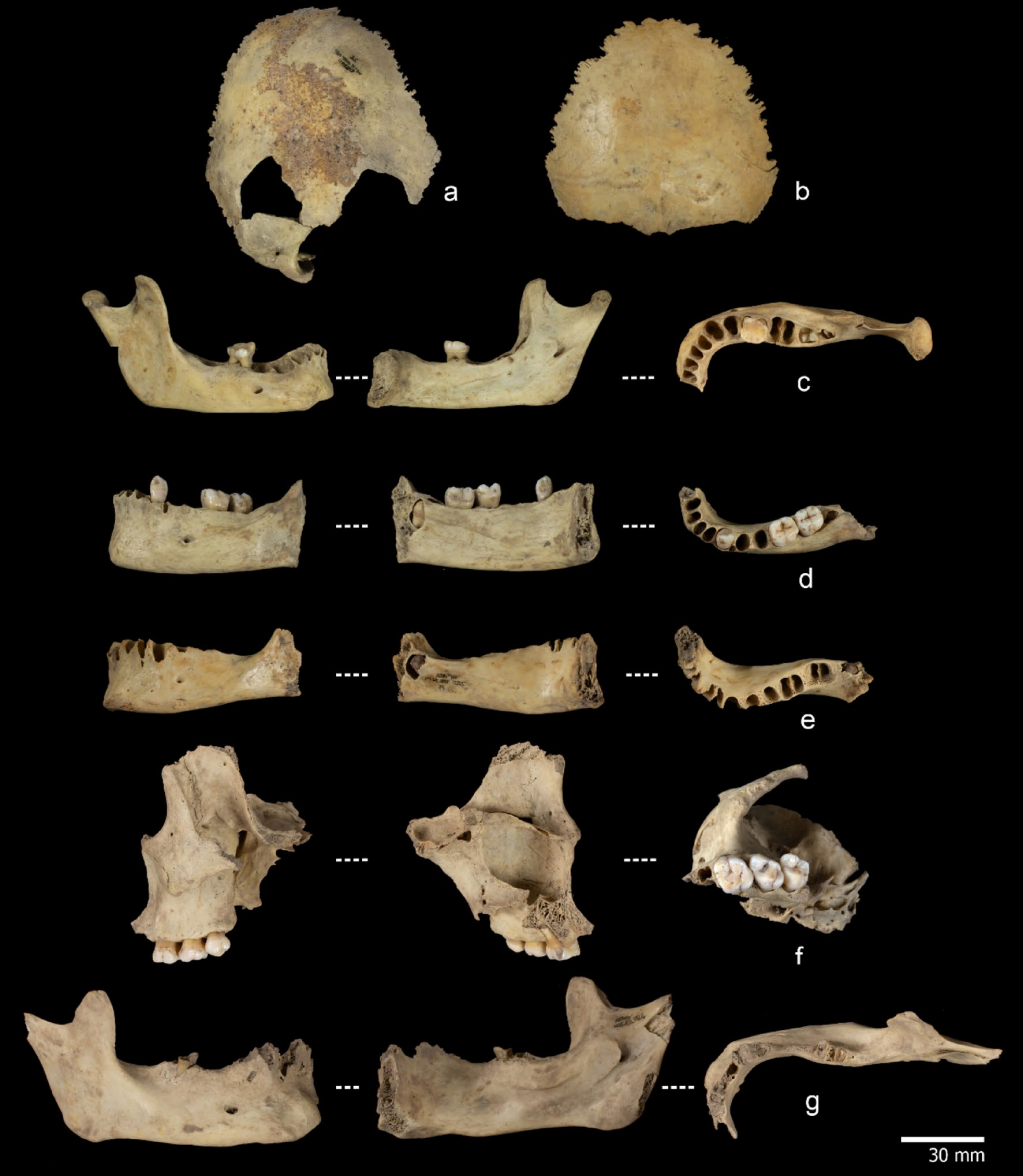
Specimens used to estimate the age at death. **(a)** Ref. ATA11-MIR105-W15-10 and ATA11-MIR105-V15-38, Pars lateralis and pars basilaris without fusion of an individual between 5 and 7 years old. **(b)** Ref. ATA15-MIR105-T14-86, Pars lateralis and pars basilaris without fusion of an individual between 5 and 7 years old**(c)** Ref. ATA16-MIR202-N37-58. Right hemimandible with Dm2 present; M1 was lost postmortem. M2 exhibits cusp coalescence, and M3 shows initial cusp formation. The estimated age at death is between 6 and 10 years. **(d)** Ref. ATA16-MIR102-S13-20. Left hemimandible with the mental eminence, canine, M1 and M2 all present, displaying minimal occlusal wear. The M3 remains in the crypt, with root formation visible: the root is shorter than the crown, and the bifurcation area is already developed. The estimated age at death is between 15 and 17 years. **(e)** Ref. ATA10-MIR202-T35-11, Left hemimandible with M3 in the crypt, displays about half of the crown formed, with dentine development in progress. The individual’s age at death was 12–15. **(f)** Ref. ATA16-MIR102-T12-21, Maxilla left of an adult individual with M1, M2, and M3 (20–25 years old). **(g)** ATA16-MIR102-T12-6, mandible with alveolar bone resorption of an individual of more than 50 years old. Photography M.D.Guillén/IPHES.

The MNI of the anthropogenically modified specimens from S100 and S200 is 11. This number was established by overlapping the femora proximal and midshafts portions (Supplementary note 2, Table S6, Figure S8) and combining that information with the age of death (Table S7). We were able to estimate the age at death of seven of the 11 individuals based on the examination of cranial and mandibular fragments (Fig. 2), including dental development, and the fusion status of the *pars lateralis* and *pars basilaris* in two occipital bones. Specifically, two individuals were under 7 years of age, one was between 10 and 11 years old, one was 14 to 16 years old, one was 15 to 17 years old, one was between 20 and 35 years of age, and finally, one was over 50 years old. The ages at death of the other two individuals could not be determined, but they were adults. The assemblage consists of a minimum of three children, two juveniles, and four adults. It should also be noted that none of the femora could be attributed to children aged 7 or younger. Therefore, two additional individuals of undetermined age, represented only by femoral remains, were included to complete the MNI.

### Burning

**Fig. 3:**
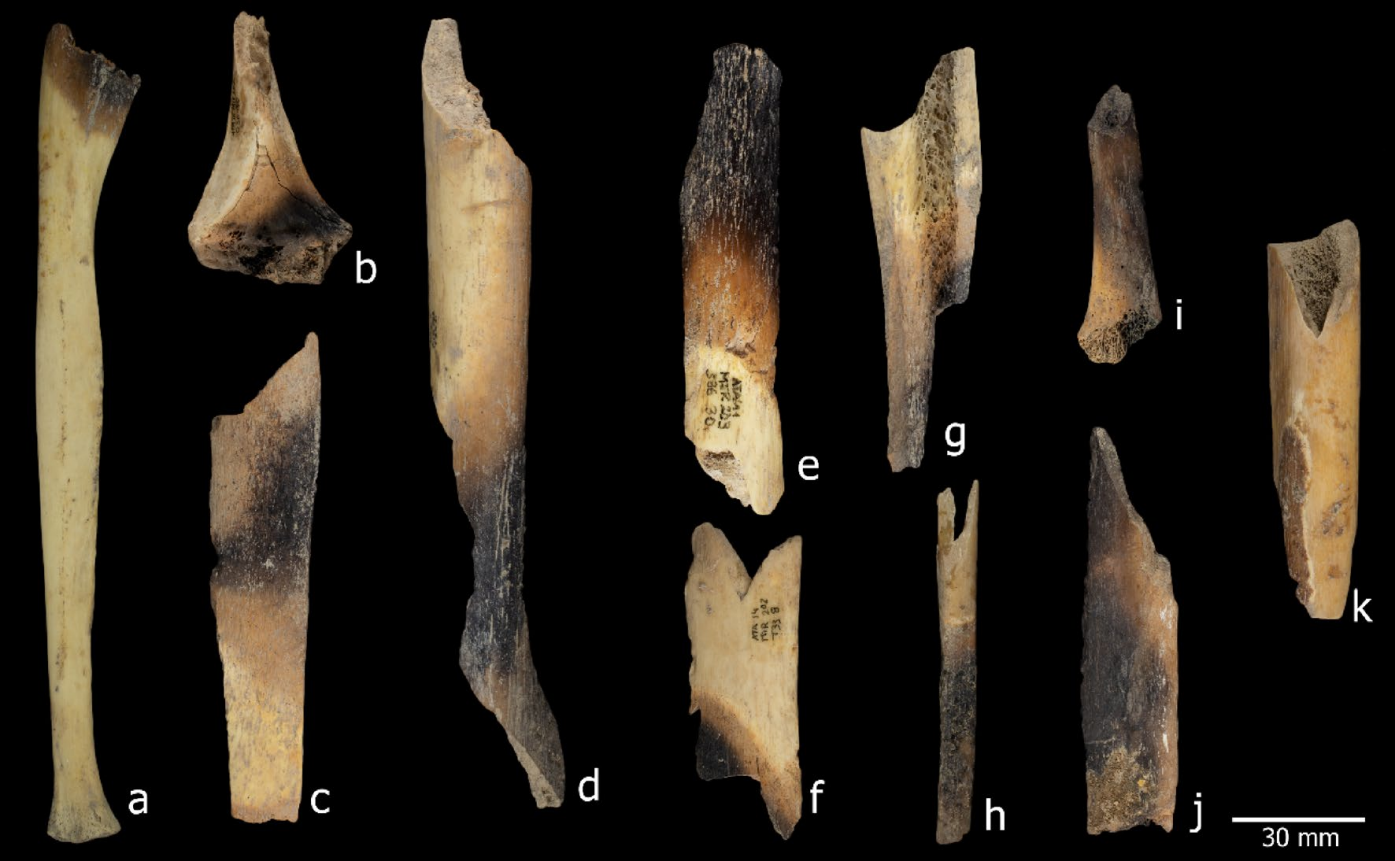
Remains modified by heating. (**a**-**j**) Burned bones showing the brown heat line of S100 and S200 of El Mirador cave. (**a**) Ref. ATA11-MIR203-S36-44, ulna. (**b**) Ref. ATA16-MI202-O36-90 distal humerus of a young. (**c**) Ref. ATA11-MIR105-V15-57, tibia midshaft. (**d**) Ref. ATA11-MIR203-T36-31, a distal shaft of tibia. (**e**) Ref. ATA11-MIR203-S36-30 humerus midshaft. (**f**) Ref. ATA16-MIR204-S36-63, femur midshaft. (**g**) Ref. ATA15-MIR202-T34-139, distal fibula. (**h**) Ref. ATA14-MIR202-T33-8, tibia midshaft. (**i**) ATA17-MIR202-O36-149, fibula. (**j**) Ref. ATA17-MIR205-P37-51, femur midshaft. (**k**) Ref. ATA09-MIR201-SC-73 femur midshaft with modifications compatible with pot polishing. Photography M.D.Guillén/IPHES.

A total of 222 human remains from S100 (NISP = 28) and S200 (NISP = 194) (Table S3) exhibited colour changes associated with cremation (Fig. 3). Within this sample of bones, on 69 (31.1%) evidence of burning was concomitant with signs of butchery (cut and/or percussion marks and/or peeling). The remaining burned bones did not exhibit any other anthropogenic modifications. The cremation of remains can result from various intentional and unintentional processes. The presence of dung layers throughout the El Mirador cave sequence^23,24^ suggests numerous dung-burning events inside the cave, during which other materials, including human remains, may have been mixed in and affected by the fire. Most of these cremations show brown and black discolorations, with only five cases found to be calcined, indicating that most specimens were not exposed to temperatures exceeding 300°C^25^. Furthermore, 41 remains exhibited a brown heat line. Symmes and collegues^25^ associate this feature with bones that were exposed to fire while still fresh and already defleshed. The fact that the bones were heated after defleshing prevents us from directly linking fire exposure to cooking practices. Although cremation may have occurred as part of the process of discarding the remains, it is impossible to confirm the relationship between the burning of the bones and the processing of the bodies.

### Pot-polishing

Signs of pot-polishing or boiling (Fig. 3k) were identified on 585 bone specimens (Table 1), characterised by slightly rounded edges and a shiny, smooth, translucent appearance. While this modification is typically recognised through qualitative criteria, experimental studies have demonstrated that macroscopic features provide a reliable basis for identification^26–28^. Butchery marks were also observed on remains exhibiting pot-polishing in both sectors, affecting 33.9% of the S100 sample and 34% of the S200 sample. Additionally, 28.7% and 25% of the remains displayed fresh fractures with curved or V-shaped outlines and oblique fracture angles^29^. The combination of these modifications suggests that this set of human bones underwent posthumous processing, including cooking.

### Cut marks

**Fig. 4:**
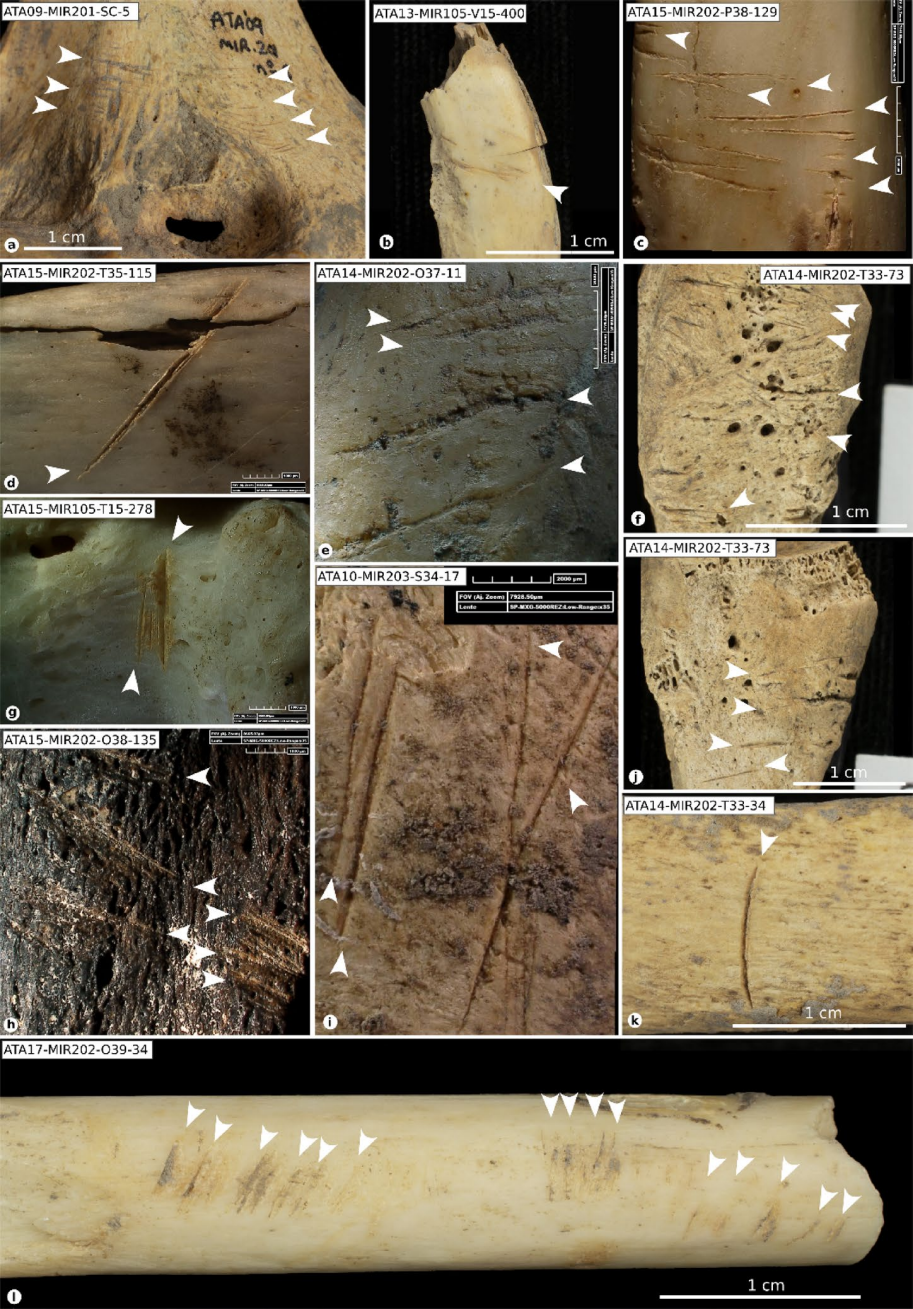
Cut marked specimens. Cut marks (white arrow) on human remains from contexts S100 and S200. (**a**) Humerus with slicing marks. (**b**, **c**) Clavicles showing slicing marks. (**d**) External side of a rib with defleshing-related slicing marks. (**e**) Proximal shaft fragment of an ulna with slicing marks associated with both disarticulation and defleshing. (**f**, **j**) Metatarsal II showing slicing marks on the proximal epiphysis. (**g**) Cervical vertebra fragment with cut marks on the articular process. (**h**) Scapular spine with cut marks. (**i**) Ilium with multiple cut marks resulting from defleshing activities. (**k**) Rib fragment with cut marks. (**l**) Fibula displaying cut marks associated with defleshing. Scale bars are shown either in detail or at the bottom of each image. Specimen numbers are indicated in white boxes. Photograpy M.D.Guillén, F. Marginedas /IPHES.

A total of 132 human remains exhibited cut marks (Fig. 4, S9), including slicing, scraping, and chop marks on various skeletal elements associated with defleshing, skinning, disarticulation, dismembering, and evisceration.

Five slicing marks were identified on skull fragments: one on a parietal bone, three on temporal bones, and one on a zygomatic bone, compatible with cutting the temporal and masseter muscles. Chop marks were found on an occipital bone near the foramen magnum, possibly related to skull fracturing. A hemi-mandible had up to 13 slicing marks and four scrape marks, suggestive of meat removal from the face and neck. Another mandible showed one slicing mark and one chop mark on the central mandibular body, while another exhibited a slicing mark near the chin. All cuts are likely related to defleshing.

Within the shoulder girdle, two clavicles had slicing marks on the posterior and inferior surfaces of the distal part, and incisions on the central portion of the superior, inferior, and anterior surfaces, related to neck meat extraction. Four scapulae exhibited one to six cut marks on the dorsal and ventral surfaces, compatible with muscle removal. Slicing marks on the coronoid process of one scapula indicate disarticulation from the humerus.

Two cervical vertebrae showed cut marks: one with transverse slices on the superior articular process related to rib disarticulation, and another with oblique incisions related to neck and upper back defleshing. Nineteen rib specimens displayed cut marks, suggesting defleshing, disarticulation, and evisceration. These included incisions and scrapes on the proximal and medial shafts, as well as slicing marks on three ribs associated with vertebrae separation. Visceral extraction was marked by slices, scrapes, and chop marks on five ribs.

One coxal bone had up to 11 slices, mainly on the gluteus maximus muscle, iliac wing, and sciatic notch, suggestive of defleshing. Another coxal bone showed cuts between the ischial tuberosity and acetabulum, related to lower limb dismemberment.

The long bones exhibited the most frequent cut marks (Table S3), with 87 fragments showing evidence of defleshing and muscle extraction from the upper and lower limbs. Slicing, chop marks, and sawing marks on 23 specimens were linked to dismemberment and disarticulation, often near the epiphyses. A chop mark on a tibia, with percussion marks, suggested deliberate bone fracturing. Three metacarpals, one metatarsal, and three phalanges also displayed cut marks, likely from the disarticulation and defleshing of the hands and feet, based on their location and experimental evidence^31^.

### Percussion marks

**Fig. 5:**
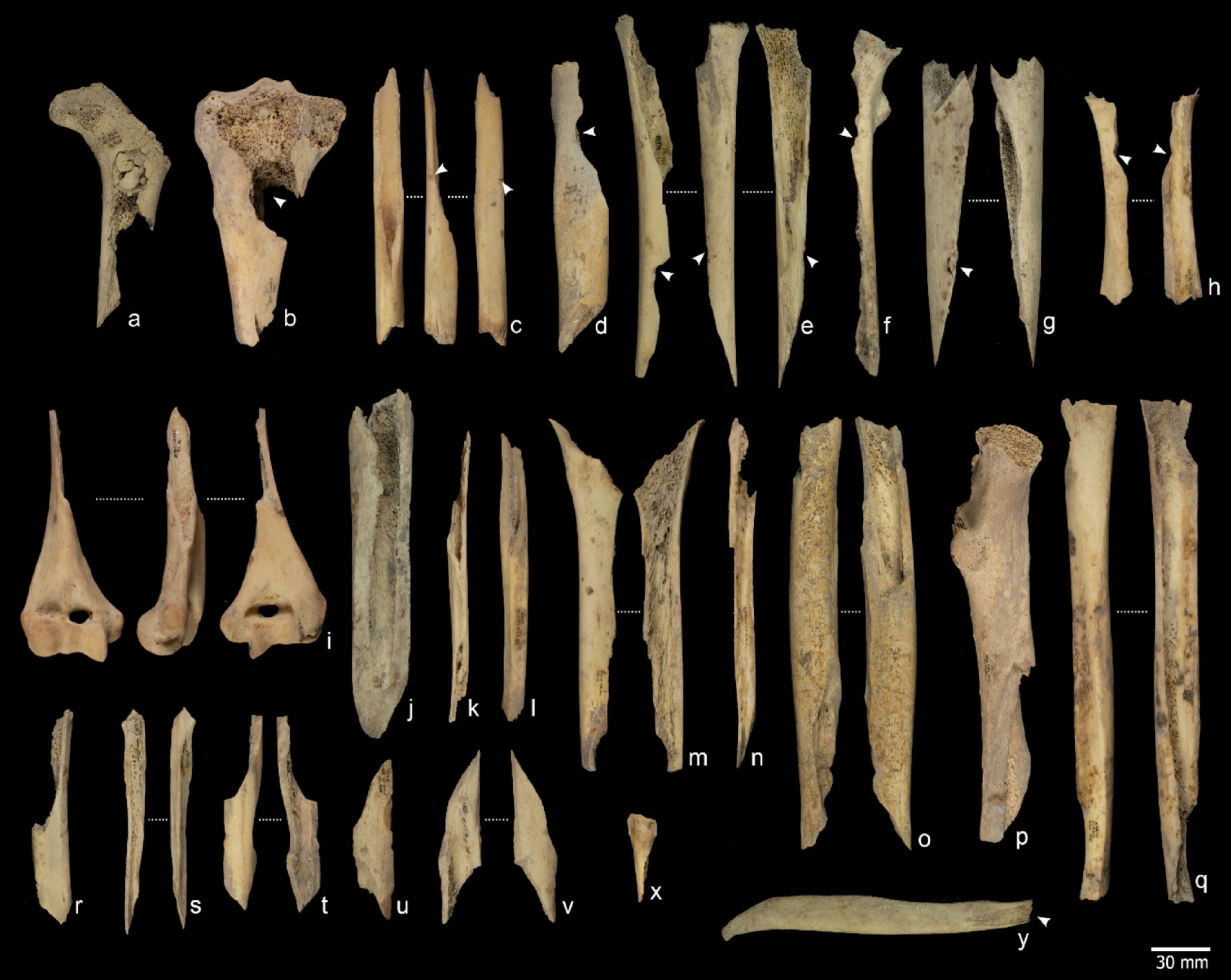
Bone with human bone breakage (A-J), fresh fractures (K-X) and peeling (Y). **(a)** Ref. ATA17-MIR202-N39-125, femur with impact percussion on the near epiphysis portion. **(b)** Ref. ATA11-MIR102-V15-10, tibia, with a big impact notch on near epiphysis portion. **(c)** Ref. ATA13-MIR202-P38-1, Tibia with chop marks related to the bone breakage. **(d)** Ref. ATA13-MIR202-P39-9, femur showing an impact notch. **(e)** Ref. ATA13-MIR203-S36-161, tibia with an impact notch. **(f)** Ref. ATA16-MIR102-T13-91notches on femur diaphysis. **(g)** Ref. ATA15-MIR102-S14-62, femur with an adhered flake and a percussion pit. **(h)** ATA15-MIR202-Q37-167 humerus with an impact notch. **(i)** Ref. ATA15-MIR105-T15-416, humerus. **(j)** Ref. ATA15-MIR102-S14-62, femur. **(k)** Ref. ATA15-MIR202-T34-52, fibula. **(l)** Ref. ATA15-MIR202-O37-124, fibula. **(m)** Ref. ATA15-MIR204-P37-39, femur. **(n)** Ref. ATA15-MIR204-P36-7, Ulna. (**o)** ATA14-MIR202-O37-86, femur. **(p)** Ref. ATA13-MIR203-R37-301, femur. **(q)** Ref. ATA10-MIR202-R35-78, tibia. **(r)** Ref. ATA09-MIR101-SC-84, femur. **(s)** Ref. ATA15-MIR204-S37-87, fibula. **(t)** Ref. ATA15-MIR202-T34-137, femur. **(u)** Ref. ATA10-MIR101-SC-325, femur. **(v)** ATA15-MIR204-P38-5, femur. **(x)** ATA10-MIR201-SC-976, II metatarsal. **(y)** ATA15-MIR202-Q38-67, rib with a classic peeling. Photography M.D.Guillén/IPHES.

A total of 245 specimens exhibited evidence of anthropogenic bone breakage through percussion techniques, including percussion pits, impact notches, and abrasions (Fig. 5). These modifications were identified on the skull fragments, long bones, mandibles, and metacarpals and metatarsals (Supplementary Note 3, Table S4), and are consistent with practices aimed at accessing marrow and brain. On crania, impact marks were observed on eight parietals, three occipitals, two temporals, one frontal, one right orbital, and four endocranial fragments. Five mandibles exhibited abrasions and pits along the mandibular body. One scapula bore a percussion pit and showed signs of peeling, likely related to shoulder disarticulation. Additionally, two ribs showed evidence of proximal percussion, also linked to disarticulation tasks.

This sample may be supplemented by 213 additional remains that exhibited features compatible with fractures occurring on fresh bones, such as curved delineations and oblique angles. However, no diagnostic modifications were observed on these bones to confidently attribute the breakage to anthropogenic activity. While this precludes definitive conclusions, the set remains relevant, as most fragments resulting from anthropogenic bone breakage lack clear surface modifications^29^. Therefore, some of these remains may still be related to marrow extraction.

### Peeling

**Fig. 6:**
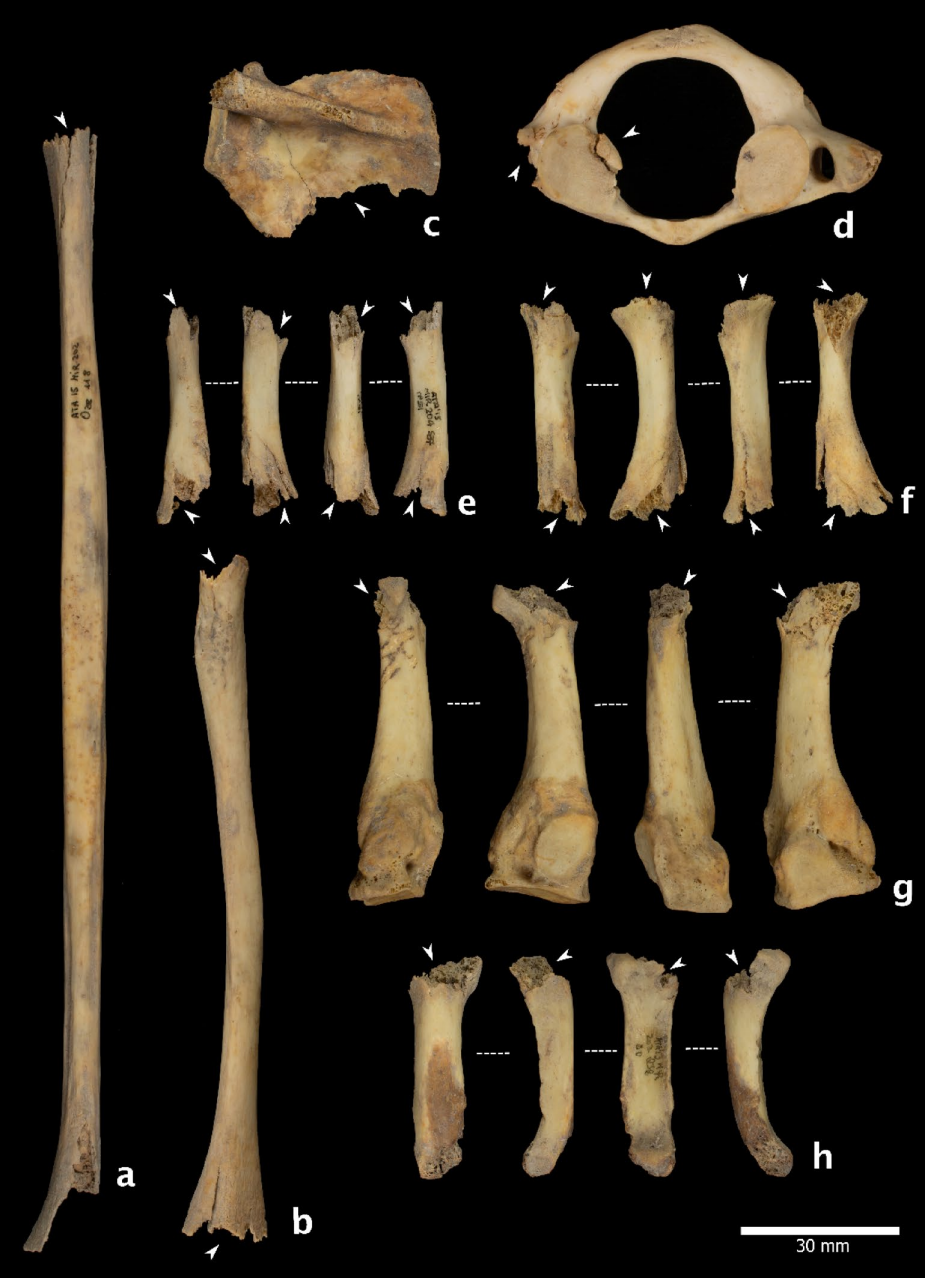
Specimens with modifications related to human chewing. **(a**) Ref. ATA15-MIR202-O38-118 fibula with scooping out on two epiphyses associated with cracks and crushing. **(b)** Ref. ATA15-MIR202-N39-37 radius of a child with scooping of the two ends and cracks. **(c)** Ref. ATA12-MIR201-P34-48, infantile scapula with a crenulated edge. **(d)** Ref. ATA09-MIR202-S35-7, atlas with tooth marks on the apophysis with double arch fractures. **(e)** Ref. ATA15-MIR204-S37-51, V metacarpal with scooping out, cracks, and crushing on the ends. **(f)** ATA15-MIR202-Q37-204, metatarsal with scooping out, cracks, and crushing on the ends. **(g)** ATA13-MIR201-O38-12, IV metatarsal with furrowing on the distal epiphysis. **(h)** ATA13-MIR202-Q38-80 proximal hand phalange. Photography M.D.Guillén/IPHES.

Peeling has been a subject of controversy, having shifted from being considered an exclusively anthropogenic modification^30^ specifically associated with human chewing and human tooth marks, to being recognised as a type of modification characterised by equifinality, meaning it can result from different agents^31^. Nevertheless, the overall taphonomic history of the samples studied contributes to determining the anthropogenic origin of this modification^31^ and whether it occurred during the butchery and/or consumption process. Peeling was observed on 43 specimens (Table S3). The most common type was classic peeling^30^ (Fig. 5y), which was observed on 27 specimens. General peeling was documented on 11 remains. Two of these specimens displayed both general and classic peeling. Two remains displayed incipient peeling. Eleven out of the 43 remains also had cut marks, and 41 exhibited pot-polishing, allowing us to attribute this modification to human manipulation. Some of these specimens (NISP = 23) bore tooth marks in the form of depressions, scores, and crushing, forming a pattern that suggests anthropogenic origin. The association of types of damage (e.g., crushing, cracks, peeling, pits, and scores) (Fig. 6) serves as evidence for attributing the tooth marks to human agents, despite ongoing debate about these types of features^30–32^.

### Human tooth marks

The identification of human tooth marks has been the focus of much debate (e.g^33^.,). However, the attribution of tooth marks to human agents can be supported by specific morphological, including crushing, longitudinal cracks, classical and incipient peeling, pits, and scores, which collectively characterise the pattern of damage resulting from human chewing^30–32^. Among the materials from S100 and S200, we identified specimens bearing tooth marks attributable to human activity. When considered alongside the taphonomic evidence detailed above, these findings provide compelling support for the occurrence of cannibalistic practices. We identified very superficial pits and scores associated with crushing, furrowing, scooping out, and peeling in the sample (NISP = 157). These modifications were mainly observed on smaller and more fragile bones, such as metacarpals and metatarsals (NISP = 41), phalanges (NISP = 27), and ribs (NISP = 19) (Fig. 5). On bones with thicker cortical surfaces (long bones, mandibles, and skull fragments), the modifications most frequently observed were grooves (NISP = 52), associated in 48% of cases with depressions. Most of the remains with identified human tooth marks also had a smooth and translucent surface related to pot-polishing (155 out of 161). Concomitance with cut marks was found on 24 occasions, 15 with percussion marks and 15 with both.

The pits measured within a metric range of between 0.58 and 2.96 mm in length and 0.43 and 3 mm in width on cortical tissue and 1.42 to 4.71 mm in length and 1.13 to 3.18 mm in width on spongy tissue, falling within the ranges of human tooth marks from experimental assemblages^31^. The 95% confidence interval of the means shows a close correspondence between the dimensions of depressions in this archaeological sample and those from experimental studies.

### Strontium

The 87Sr/86Sr isotope analysis samples were obtained from the femoral remains of five individuals. The 87Sr/86Sr ratios ranged from 0.709169 to 0.710587 (Table 1). Mean values of 0.709–0.710 are associated with the Cenozoic basin. The Sierra de Atapuerca is at the north-eastern end of the Cenozoic Duero River Basin. According to predictive 87Sr/86Sr isoscape models for the map of the Iberian Peninsula^34^ the five individuals with strontium isotope ratios falling between 0.709 and 0.7099 may be of local origin. However, it should be noted that the area extends towards the west in the predictive model.

**Table 1:** 87Sr/86Sr results for the tested samples.

Lab ID	Reference	Bone identification	^87^Sr/^86^Sr Raw	± 95%CI	^87^Sr/^86^Sr Adjusted*	± 95%CI
IS-1230	ATA2016-MIR204-O38-6	Femur	0,709808	0,000017	0,709814	0,000017
IS-1231	ATA2015-MIR204-Q37-11	Femur	0,709162	0,000096	0,709169	0,000096
IS-1232	ATA2015-MIR204-P37-39	Femur	0,710581	0,000138	0,710587	0,000138
IS-1233	AA2013-MIR202-P39-9	Femur	0,709721	0,000019	0,709727	0,000019
IS-1234	ATA2012-MIR203-S37-13	Femur	0,709759	0,000072	0,709765	0,000072

*Adjusted relative to the accepted value of 0.710248 ± 0.000003 (MacArthur et al., 2001) for SRM 987.

## Discussion

Prehistoric human cannibalism has been increasingly recognised within the European archaeological record in recent years. Significant advancements in the analyses of these assemblages have been made possible by the application and implementation of taphonomic methods to the study of Pleistocene and early Holocene human remains. Current observations suggest an increase in assemblages with anthropogenic modifications in Europe towards the end of the Upper Palaeolithic and Mesolithic^36–39^ and throughout the Neolithic among the earliest farming communities^15,40–42^. The archaeological findings in level MIR4 in the central pit of El Mirador cave are among the most recent discoveries of this type. The manufacture of skull cups, perhaps for ceremonial purposes, was also documented^19^. During posterior excavations at the site, new human remains in different archaeological contexts were recovered, with one assemblage containing specimens with cultural modifications such as cut and percussion marks^17^.

The localization of a human-modified sample intermixed among the collective burial in S200, or the livestock pen remains in S100 forces us to consider and discuss alternative hypotheses to cannibalism since these modifications may have arisen from activities unrelated to butchering processes and the consumption of the deceased. Cultural taphonomic signs on human remains can stem from a variety of behaviours. Firstly, funerary rituals in which the removal of skulls and/or the disarticulation of other elements for secondary deposition are often identified through slicing and scrape marks^43–45^. However, these cuts are less frequent than when related to the preparation of bodies for consumption, typically affecting no more than 1 to 5% of the remains. Secondly, similar slice and chop marks can also be produced by injuries caused by stone, metal, or bone weapons or in aggressive encounters^46,47^. Cuts related to violent injuries are mainly, although not exclusively, located on the ribs and vertebrae, with a prevalence of chop marks over slicing marks. Between the resulting modifications of forceful encounters, cut marks, perimortem fractures, and limb mutilations can also be present. The types of cuts, their incidence, and their possible connection to depressed fractures on cranial vaults make it possible to distinguish between damage caused by violence and modifications produced during the butchering process.

Nevertheless, when mixed with signs of cannibalism, they can be difficult to discern^48^. However, when modifications resulting from violent encounters and those produced during human processing appear together within the same assemblage, distinguishing between them becomes particularly challenging. In such cases, cut marks and depressed fractures can be further obscured by defleshing, disarticulation, and subsequent bone breakage. Third, cut marks concentrated on specific anatomical areas may indicate the removal of body parts for war trophies. Scalping is especially relevant, producing a distinctive pattern of incisions encircling the skull, from the forehead, over the ears, to the back of the head^49^. Scalping may also occur as part of the butchering process during the preparation of the body for consumption^27,29^ although with a broader distribution involving the removal of skin, ears, and flesh. When combined with the production of skull cups, these practices leave a recognisable arrangement of slicing and scraping marks, often parallel to or encircling the sagittal suture^17^.

The pattern of modifications found on the modified Neolithic human bones of El Mirador cave is inconsistent with these three possible scenarios. Instead, the evidence supports a comprehensive butchering process involving meat, viscera, bone marrow, and brain extraction. Cut marks on these specimens are abundant, indicating extensive processing rather than the removal of skeletal elements for secondary burial, injuries sustained during interpersonal conflict, or practices related to trophy acquisition. The presence of fresh bone breakage, burning, pot polishing, and human tooth marks reflects a consistent pattern of modifications. Taken together, this taphonomic evidence closely matches the indicators documented in other prehistoric assemblages interpreted as cases of cannibalism^16,27,28,33,36,42,43,50–52^.

The interpretation of the sample is further strengthened by radiocarbon dating, which has contributed crucial data for its interpretation. Dating results consistently point to a specific timeframe in the late Neolithic period, adding to the evidence that the anthropogenic modifications at El Mirador occurred across different phases of cave use, including the Bronze Age cannibalism episode documented in Level MIR4. The evidence of Neolithic cannibalism at El Mirador Cave, along with its broader intensification in Europe around 6,000 years BP, suggests that this practice was becoming increasingly common during this period. This intensification of cannibalistic behaviour is likely due to a variety of factors, making it challenging or impossible to identify a single underlying motive. Of all the classifications of cannibalism reported in anthropological and archaeological studies^27,51^we propose three potential behavioural contexts that should be considered in the case of the new Neolithic sample from El Mirador cave: funerary traditions, famine periods, and conflicts culminating in intergroup confrontations. The first of these entails the consumption of a population’s deceased by their relatives as part of some type of ritual, usually connected to ideas relating to the regeneration of bodily fluids^53^. The funerary scenario should be taken into consideration, given that the population of cannibalised individuals was of local or regional origin. However, the highly localised and temporally brief nature of the episode appears inconsistent with patterns expected from ritual endocannibalism, which are typically more recurrent. This has led us to assign greater weight to the other two interpretive alternatives.

The alternative of a famine scenario in El Mirador cave is more complex and must be approached starting with a review of the paleoenvironmental data. The environmental record, including plant and micromammal remains from El Mirador cave, reflects a progressive trend towards increased aridity in the area surrounding the Sierra de Atapuerca, beginning around 6,000 years BP and reaching a peak during the Bronze Age^53,54^. In levels MIR6 to MIR9, where the latest Neolithic occupations in the cave were documented, the presence of *Quercus*, especially the evergreen species, increases, clearly indicating a decline in deciduous leaf values and pointing to drier environmental conditions in line with the general climatic conditions in Europe at that time. However, palynological and anthracological analyses indicate that although the landscape underwent changes due to a possible decrease in rainfall, favouring less-demanding taxa regarding water requirements, the general environmental context at the time was a typically Mediterranean mixed forest^54,55^. Evidence points to the combined management of forest resources and agricultural practices can be observed^56^ and although this evidence is not conclusive, no clear signs of food scarcity have been identified during the documented occupations. The diverse diet and availability of a wide range of food sources would have ensured adequate nutrition for the agropastoral communities of El Mirador cave, as evidenced by the low incidence of metabolic pathologies typically associated with nutritional stress^57^. However, caution is warranted when interpreting this evidence in relation to the type of cannibalism practised at the site, particularly if the individuals consumed did not belong to the same group as the consumers. Furthermore, it is also important to exercise caution, as the circumstances leading to famine are not always the result of long-term environmental trends accessible through archaeological proxies. Short-term, high-impact events—such as crop failures, severe winters, or parasitic outbreaks—may leave little to no detectable trace in the archaeological record, despite their potentially significant impact on prehistoric communities.

A common feature of starvation periods is increased mortality among the most vulnerable individuals, primarily the elderly and children^58^. The age structure of the anthropogenically modified individuals, consisting of at least three children, two juveniles, and four adults, including one individual over 50 years old, does not suggest a mortality profile dominated by the most vulnerable, as might be expected in a starvation scenario. While the presence of children and an elderly individual could align with stress-related attritional mortality, the overall distribution is more balanced. In our case, the absence of a demographic skew toward young children or older adults, combined with the archaeological context, makes it difficult to confidently associate the age profile with a famine episode. A similar age pattern is observed at Neolithic sites in central Europe, where mass violent events involving entire communities or family groups have been recorded^59,60^. Recent paleodemographic studies, such as those on the Nordic Corded Ware population, have shown that epidemic or conflict-related stressors often result in high, age-nonspecific mortality, frequently including large numbers of subadults^61^. Demographic compositions comparable to that observed at El Mirador have also been recorded at Early Neolithic massacre sites such as Asparn/Schletz (Austria), and Talheim and Schöneck-Kilianstädten (Germany), where archaeological and osteological evidence demonstrates the systematic killing of both adults and children^59^. A massacre has also been identified at the Pyrenean site of Els Trocs (Spain); the recovered individuals include both children and adults, supporting the interpretation that the victims belonged to a socially cohesive group, possibly an extended household^62^.

So, several traits of the El Mirador assemblage are consistent with a similar interpretation. These include the short-term depositional episode coinciding with the final Neolithic occupation phase (Fig. 2), the interruption of livestock-related activities, and the cave’s functional shift, from pen to funerary space, in the subsequent Chalcolithic period. Together, these features lend weight to the hypothesis of ‘warfare cannibalism,’ in which the victims, likely a nuclear or extended family, may have been killed in a single event by a neighbouring or external group. Although no perimortem trauma has been identified on the remains, this does not invalidate the hypothesis, as many lethal injuries leave no skeletal trace. Moreover, in cases where injuries caused by interpersonal violence and cannibalistic processing overlap, it is often difficult to distinguish butchering from killing^59^.

Comparable interpretations have been proposed at sites like Herxheim (Germany), where hundreds of human remains were deposited and processed, possibly during ritualised cannibalistic feasts^15^. While originally interpreted as ceremonial, later studies suggest the possibility of violence, as evidenced by perimortem cranial injuries and connections to LBK-period massacres^33^. Fontbrégoua (France) also presents evidence of cannibalism interpreted within a conflict-related framework, with the absence of hands and feet suggesting the collection of war trophies^63^. In contrast, no such patterns have been identified at El Mirador, nor have cause-of-death injuries been observed.

Ethnographic and historical records show that, outside starvation contexts, cannibalism often reflects deeply embedded symbolic, spiritual, or social beliefs^64^. In fact, it is common for groups that engage in funerary endocannibalism to also practice warfare-related exocannibalism, with both forms embedded in symbolic and ritual frameworks^12–14,64^. Nevertheless, it is impossible to infer institutionalized behaviour at El Mirador or other Iberian Neolithic or Bronze Age sites, as the cannibalism events appear to be isolated episodes that lack evidence of continuity.

Although still speculative, the hypothesis that the El Mirador assemblage reflects a case of conflict-related cannibalism is consistent with a growing body of archaeological evidence pointing to widespread inter-group violence during the Neolithic. This period is increasingly recognised as one marked by conflict and instability, driven by profound social and demographic transformations linked to the shift from foraging to farming. High population pressure, competition over resources, and interactions between indigenous Mesolithic groups and incoming Neolithic farming communities often resulted in violent confrontations of varying scale^59,60]^^65–68^. This pattern of conflict continued into the Chalcolithic and intensified in the Bronze Age^69^.

In the Iberian Peninsula, the archaeological record reveals a wide spectrum of violence, ranging from small- and medium-scale clashes^46,62,70^ to massacres of entire communities^71,72^. These conflicts likely stemmed from territorial disputes or tension between neighbouring groups and newly arrived populations. However, skeletal trauma, often poorly preserved or underreported, only provides a minimal estimate of the actual degree of violence. Despite these limitations, endemic conflict is now recognised as a defining feature of early European farming societies^65^.

Within this broader context, the findings from El Mirador Cave suggest that cannibalism may fall within the same spectrum of violent practices, possibly reflecting the deeper social tensions and conflict dynamics characteristic of Neolithic and Chalcolithic communities.

## Methods

A total of 5,056 human remains from the Central Pit, S100, and S200 of El Mirador cave were examined. Detailed descriptions were recorded for all specimens displaying anthropogenic modifications, including the bone element, the lateralization (right or left), the preserved portion, and the view. Dental development^73^ as well as the degree of cranial suture fusion^74^ were used to estimate individuals’ age at death.

Cut marks were identified based on the proposals of Potts and Shipman^75^ and the morphological criteria of Domínguez-Rodrigo et al.^76^. The complete surface of each bone was examined using a stereomicroscope (OPTHEC 120HZ) with magnifications ranging from 10 to 40 with direct and oblique lighting. Three types of cut marks were recognized: slice, scrape, and chop marks. Slice marks occur when force is applied with the tool’s edge parallel to the bone’s surface, scrape marks result from tool being dragged in a direction perpendicular to the bone’s surface, and chop marks are produced by the application of dynamic force (percussion) with a sharp or blunt edge^77^. In the analysis of the cut marks, we considered the number of individual striations, their location on the skeletal element, and their orientation relative to the bone’s longitudinal axis (oblique, longitudinal, transversal). This description allowed us to establish butchery practices based on the observations of White^27^ and Saladié and collegues^29^. The presence/absence and location of percussion marks, signs of impact, conchoidal scars, abrasions, and peeling were also recorded on each of the specimens studied^27,78^. Fracture surfaces were analysed following Villa and Mahieu’s^79^ criteria to distinguish fresh breaks characterized by curved and V-shaped delineations with oblique angles from dry breaks with transverse fracture surfaces and right angles. Peeling was described as general (an area of the whole dorsal or ventral cortex of a rib is peeled back for some length, revealing the internal trabeculae), classic (characterized by a rough surface with parallel grooves that remain as the two halves separate), or incipient (strip of lamella is only partially peeled back against the rib shaft, not fully removed from the specimen)^30^.

Additionally, bones exhibiting a smooth, shiny, and translucent surface were recorded, as these characteristics suggest bone boiling. Cremation was described based on the colours (brown, black, grey, white) of the bones and their combination on the surface^80^. Human tooth marks were identified considering their location on the bones, morphology, and combination of modifications^31^. Within the assemblage, punctures, pits, scores, furrows, scooping-out, crushing, and cracks were identified. Comparisons were made based on the type of tissue (cancellous or cortical) in which the marks were found. When the criteria for assignment were not met, the actor responsible for the tooth mark was considered indeterminate, and the specimens were excluded from the described results.

Eight human remains exhibiting anthropogenic modifications (cut marks, percussion marks, or both) were selected for radiocarbon dating. We also considered the stratigraphic distribution of the specimens in the selection process to ensure the analysis of at least one bone from each level, including those with reworked sediments. A Bayesian model was constructed using OxCal 4.4 software^81^ and the calibrated IntCal20 curve^82^ to refine radiocarbon date calibrations and detect any discrepancies between the 14 C ages and their relation to other radiocarbon dates from the three excavated deposits in El Mirador cave.

Five bones were sampled and analysed by Isobar Science using 87Sr/86Sr isotopes. Strontium was isolated from the samples by extraction chromatography and analysed by MC-ICO-MS (Thermo Fisher Neotune PlusTM) following the methods described by Pourmand et al.^82^ and Pourmand and Dauphas^83^ The measured 87Sr/86Sr ratios were corrected for mass bias and isobaric interference. The final ratio was further adjusted relative to the accepted value of 0.710248 ± 0.00000387 for the SRM 987, allowing for comparison with literature measurements of radiogenic Sr isotopes.

## Electronic supplementary material

Below is the link to the electronic supplementary material.

Supplementary Material 1

## Data Availability

All data needed to evaluate the conclusions in the paper are present in the paper and/or the Supplementary Materials.

## References

[1] Weiss-Krejci, E. The formation of Mortuary deposits. In *Social Bioarchaeology* (eds. Agarwal, S.C & Glencross, B. A.) 68–106 (Wiley-Blackwell, 2011).

[2] Garrido, R., Rojo, M., Tejedor, C. & García-Martínez, I. Las máscaras de la muerte: ritos funerarios en el Neolítico de la Península Ibérica. in *El Neolítico en la Península Ibérica y su contexto europeo* (eds. Rojo Guerra, M., Garrido Pena, R. & García Martínez de Lagrán, I.) 143–174 (2012).

[3] Blasco, A., Edo, M., Villalba, M. J. & Saña, M. Primeros datos sobre la utilización sepulcral de la Cueva de Can Sadurní (Begues, Baix Llobregat) en el Neolítico Cardial. in *III Congreso del Neolítico en la Península Ibérica* (eds. Arias, P., Ontañon, R. & García-Moncó, C.) 867–878 (2005).

[4] Varela-Gomes, M. Castelo Belinho (Algarve): a ritualização funerária em meados do V milénio AC. in *Os últimos caçadores-recolectores e as primeiras comunidades produtoras do sul da Península Ibérica e do norte de Marrocos: actas do workshop (Faro 2–4 Novembro de 2009)* (eds. Gibaja, J. F. & Carvalho, A. F.) 69–79 (Universidade do Algarve Faro, 2010).

[5] Molist, M. et al. Los orígenes del megalitismo en Cataluña en el marco de las prácticas funerarias del Neolítico. in *Actas del Congreso Internacional sobre Megalitismo y otras manifestaciones funerarias contemporáneas en su contexto sociconómico y cultural. Munibe (suplemento)* (eds. Fernández-Eraso, J. & Mujika, J. A.) vol. 32 212–224 (2010).

[6] Bettencourt, A. M. S. La edad Del bronce En El Noroeste de La Península ibérica: Un análisis a partir de Las prácticas funerarias. *Trab Prehist*. **67**, 139–173 (2010).

[7] Milesi García, L. et al. Funerary practices in megalithic tombs during the Argaric bronze age in South-Eastern iberia: the cemetery of Los Eriales. *J. Archaeol. Sci. Rep.* **49**, 103972 (2023).

[8] Delibes, G., Alonso, O., Estremera, M. S. & Pastor, J. F. ¿Sepultura o reliquia? A propósito de Un Cráneo Hallado En ambiente habitacional En La Cueva de La vaquera (Segovia). *SAGVNTVM Extra*. **2**, 429–434 (1999).

[9] Acosta Martínez, P. Las culturas Del Neolítico y Calcolítico En Andalucía occidental. *Espac Tiempo Forma Ser. Prehist Arqueol*. 10.5944/etfi.8.1995.4720 (2013).

[10] García-Rivero, D. et al. The exceptional finding of locus 2 at Dehesilla cave and the middle neolithic ritual funerary practices of the Iberian Peninsula. *PLoS One*. **15**, e0236961 (2020). 32790702 10.1371/journal.pone.0236961 PMC7425899

[11] Pettitt, P. *The Palaeolithic Origins of Human Burial* (Routledge, 2010).

[12] Conklin, B. A. *Consuming Grief: Compassionate Cannibalism in an Amazonian Society* (University of Texas, 2001).

[13] Fausto, C. Feasting on people eating animals and humans in Amazonia. *Curr. Anthropol.* **48**, 497–529 (2007).

[14] Viláça, A. Relations between funerary cannibalism and warfare cannibalism: the question of predation. *Ethnos* **65**, 83–106 (2000).

[15] Boulestin, B. et al. Mass cannibalism in the linear pottery culture at Herxheim (Palatinate, Germany). *Antiquity* **83**, 968–982 (2009).

[16] Cáceres, I., Lozano, M. & Saladié, P. Evidence for bronze age cannibalism in El Mirador cave (Sierra de atapuerca, burgos, Spain). *Am. J. Phys. Anthropol.* **133**, 899–917 (2007). 17492670 10.1002/ajpa.20610

[17] Marginedas, F. et al. Making skull cups: butchering traces on cannibalised human skulls from five European archaeological sites. *J. Of*. **114**, 105076 (2020).

[18] Vergès, J. M. Funerary practices at El Mirador cave. In *Prehistoric Herders and Farmers: A Transdisciplinary Overview To the Archeological Record from El Mirador Cave* (eds Allué, E. et al.) 131–146 (Springer International Publishing, 2022).

[19] Vergès, J. M. et al. El Mirador cave: biogeographical setting and site description. In *Prehistoric Herders and Farmers: A Transdisciplinary Overview To the Archeological Record from El Mirador Cave* (eds Allué, E., Martín, P., Vergès, J. M. et al.) 13–34 (Springer International Publishing, 2022).

[20] Martín, P. et al. Springer International Publishing, Cham,. Husbandry and Wild Animal Exploitation. Characteristics and Evolution from a Multidisciplinary Perspective. in *Prehistoric Herders and Farmers: A Transdisciplinary Overview to the Archeological Record from El Mirador Cave* (eds. Allué, E., Martín, P. & Vergès, J. M.) 225–250 (2022).

[21] Allué, E., Martín, P. &amp; Vergès, J. M. *Prehistoric Herders and Farmers: A Transdisciplinary Overview To the Archeological Record from El Mirador Cave* (Springer Nature, 2022).

[22] Vergès, J. M. et al. Los niveles neolíticos de la Cueva del Mirador (Sierra de Atapuerca, Burgos): nuevos datos sobre la implantación y el desarrollo de la economía agropecuaria en la submeseta norte. in *IV Congreso del Neolítico Peninsular* (eds. Hernández Pérez, M., Soler Díaz, J. A. & López Padilla, J. A.) 418–427 (Museo Arqueológico de Alicante, Alicante, (2008).

[23] Burguet-Coca, A. et al. The fumier sequences of El mirador: an approach to fire as a Sociocultural practice and taphonomic agent. In *Prehistoric Herders and FarmersA Transdisciplinary Overview To the Archeological Record from El Mirador Cave* (eds Allué, E., Martín, P., Vergès et al.) 89–110 (Springer Cham, 2022).

[24] Angelucci, D. E., Boschian, G., Fontanals, M., Pedrotti, A. & Vergès, J. M. Shepherds and karst: the use of caves and rock-shelters in the mediterranean region during the neolithic. *World Archaeol.* **41**, 191–214 (2009).

[25] Symes, S. A. et al. Thermal alterartions to bone. In *Manual of Forensic Taphonomy* (eds Pokines, J. T., Symes, S. A. et al.) 367–402 (CRS, 2013).

[26] Bosch, P., Alemán, I., Moreno-Castilla, C. & Botella, M. Boiled versus unboiled: a study on neolithic and contemporary human bones. *J. Archaeol. Sci.* **38**, 2561–2570 (2011).

[27] White, T. D. *Prehistoric Cannibalism at Mancos 5MTUMR-2346* (Princenton Univeristy., 1992).

[28] Turner, C. G., Turner, J. A. & II & *Man Corn. Cannibalism and Violence in the Prehistoric American Southwest* (University of Utah, 1999).

[29] Saladié, P. et al. Experimental butchering of a chimpanzee carcass for archaeological purposes. *PLoS One*. **10**, e0121208 (2015). 25793521 10.1371/journal.pone.0121208 PMC4368797

[30] Pickering, T. R. et al. Taphonomy of ungulate ribs and the consumption of meat and bone by 1.2-million-year-old hominins at Olduvai gorge, Tanzania. *J. Archaeol. Sci.* **40**, 1295–1309 (2013).

[31] Saladié, P., Rodríguez-Hidalgo, A., Díez, C., Martín-Rodríguez, P. & Carbonell, E. Range of bone modifications by human chewing. *J. Archaeol. Sci.* **40**, 380–397 (2013).

[32] Fernández-Jalvo, Y. & Andrews, P. When humans Chew bones. *J. Hum. Evol.* **60**, 117–123 (2011). 20951407 10.1016/j.jhevol.2010.08.003

[33] Boulestin, B. & Coupey, A. S. *Cannibalism in the Linear Pottery Culture: the Human Remains from Herxheim* (Archaeopress Publishing Limited, 2015).

[34] Díaz-del-Río, P. et al. Paleomobility in iberia: 12 years of strontium isotope research. *J. Archaeol. Science: Rep.* **46**, 103653 (2022).

[35] Boulestin, B. Approche Taphonomique des restes humaines. Le Cas des mésolithiques de La Grotte des Perrats et Le problème du cannibalisme En préhistoire récente Européenne. *BAR Int. Ser.* **776**, 276 (1999).

[36] Bello, S. M., Saladié, P., Cáceres, I., Rodríguez-Hidalgo, A. & Parfitt, S. A. Upper palaeolithic ritualistic cannibalism at gough’s cave (Somerset, UK): the human remains from head to toe. *J. Hum. Evol.* **82**, 170–189 (2015). 25887278 10.1016/j.jhevol.2015.02.016

[37] Marsh, W. A. & Bello, S. Cannibalism and burial in the late upper palaeolithic: combining archaeological and genetic evidence. *Quat Sci. Rev.* **319**, 108309 (2023).

[38] Morales-Pérez, J. V. et al. Funerary practices or food delicatessen? Human remains with anthropic marks from the Western mediterranean mesolithic. *J. Anthropol. Archaeol.* **45**, 115–130 (2017).

[39] Marginedas, F. et al. New insights of cultural cannibalism amongst Magdalenian groups at Maszycka cave, Poland. *Sci. Rep.* **15**, 2351 (2025). 39915582 10.1038/s41598-025-86093-w PMC11802845

[40] Sala, N. & Conard, N. Taphonomic analysis of the hominin remains from Swabian Jura and their implications for the Mortuary practices during the upper paleolithic. *Quat Sci. Rev.* **150**, 278–300 (2016).

[41] Botella, M. C. Restos Humanos Eneolíticos Con incisiones En La provincia de Granada. *Anales Del. Desarrollo*. **17**, 401–423 (1973).

[42] Solari, A., Botella, M. & Alemán, I. *Canibalismo En La Cueva De Malamuerzo: Identificación De Huellas De Manipulación Intencional En Restos Óseos Humanos De Origen Arqueológico (Granada, España)* (Archaeo, 2012).

[43] Santana, J., Rodríguez-Santos, F. J., Camalich-Massieu, M. D., Martín-Socas, D. & Fregel, R. Aggressive or funerary cannibalism? Skull-cup and human bone manipulation in Cueva de El Toro (Early neolithic, Southern Iberia). *Am. J. Phys. Anthropol.* **169**, 31–54 (2019). 30802307 10.1002/ajpa.23805

[44] Robb, J. et al. Cleaning the dead: neolithic ritual processing of human bone at scaloria cave, Italy. *Antiquity* **89**, 39–54 (2015).

[45] Bello, S. M., Wallduck, R., Dimitrijević, V., Živaljević, I. & Stringer, C. B. Cannibalism versus funerary defleshing and disarticulation after a period of decay: comparisons of bone modifications from four prehistoric sites. *Am. J. Phys. Anthropol.* **161**, 722–743 (2016). 27561127 10.1002/ajpa.23079

[46] Moreno-Ibáñez, M. Á. et al. Death in the high mountains: evidence of interpersonal violence during late chalcolithic and early bronze age at Roc de les Orenetes (Eastern pyrenees, Spain). *Am. J. Biol. Anthropol.* **184**, e24909 (2024). 38415956 10.1002/ajpa.24909

[47] Sparacello, V. S. et al. Projectile weapon injuries in the Riparo Tagliente burial (Veneto, Italy) provide early evidence of late upper paleolithic intergroup conflict. *Sci. Rep.* **15**, 14857 (2025). 40295539 10.1038/s41598-025-94095-x PMC12037905

[48] Lambert, P. M. Patterns of violence in prehistoric hunter-gatherer societies of coastal Southern California. In *Troubled Times* Vol. 3 (eds Martin, D. L. & Frayer, D. W.) 77–109 (Gordon and Breach, 1997).

[49] Olsen, S. L. & Shipman, P. Cutmarks and perimortem treatment of skeletal remains on the Northern plains. In *Skeletal Biology in the Great Plains: Migration, Warfare, Health, and Subsistence* (eds Owsley, D. W. & Jantz, R. L.) 377–387 (Smithsonian Institution, 1994).

[50] Saladié, P. & Rodríguez-Hidalgo, A. Archaeological evidence for cannibalism in prehistoric Western europe: from Homo antecessor to the bronze age. *J. Archaeol. Method Theory*. **24**, 1034–1071 (2017).

[51] Saladié, P. et al. Intergroup cannibalism in the European early pleistocene: the range expansion and imbalance of power hypotheses. *J. Hum. Evol.* **63**, 682–695 (2012). 22944348 10.1016/j.jhevol.2012.07.004

[52] Bañuls-Cardona, S. & Bisbal-Chinesta, J. F. Small Vertebrate Accumulations from El Mirador Cave: A Climate and Ecological Analysis. in *Prehistoric Herders and Farmers: A Transdisciplinary Overview to the Archeological Record from El Mirador Cave* (eds. Allué, E., Martín, P. & Vergès, J. M.) 57–85Springer International Publishing, Cham, (2022).

[53] Expósito, I., Allué, E. & Burjachs, F. Vegetation and Climate at El Mirador Cave: Exploring the Beginning of Cultural Landscapes. in *Prehistoric Herders and Farmers: A Transdisciplinary Overview to the Archeological Record from El Mirador Cave* (eds. Allué, E., Martín, P. & Vergès, J. M.) 35–55Springer International Publishing, Cham, (2022).

[54] Expósito, I., Burjachs, F. & Vergès, J. M. Human trace on the landscape during the holocene at El Mirador cave (Sierra de atapuerca, Spain): The palynological evidence. *Holocene* **27**, 1201–1213 (2017).

[55] Cano-Cano, N., Burguet-Coca, A., Euba, I., Expósito, I. &amp; Allué, E. Forest Management and Agriculture Practices at El Mirador Cave. *Interdisciplinary Contributions to Archaeology* 271–293 Preprint at (2022). 10.1007/978-3-031-12278-1_14

[56] Iglesias-Bexiga, J., Yustos, M. & Etxeberria-Gabilondo, F. Life and death in El Mirador cave. Anthropological and palaeopathological analysis of a collective burial. In *Prehistoric Herders and Farmers: A Transdisciplinary Overview To the Archeological Record from El Mirador Cave* (eds Allué, E. et al.) 147–166 (Springer International Publishing, 2022).

[57] Geber, J. Interring ‘deserving’ child: archaeology deaths burials children Kilkenny workhouse during great famine Ireland. In *Children, Death and Burial: Archaeological Discourses* (ed. Murphy, E.) 1845–1852 (Oxbow Books, 2017) (**& Le Roy, M.)**).

[58] Meyer, C., Lohr, C., Gronenborn, D. & Alt, K. W. The massacre mass grave of Schöneck-Kilianstädten reveals new insights into collective violence in Early Neolithic Central Europe. *Proc. Natl. Acad. Sci. U. S. A.* 112, 11217–11222 (2015). 10.1073/pnas.1504365112 PMC4568710 26283359

[59] Schroeder, H. et al. Unraveling ancestry, kinship, and violence in a late neolithic mass grave. *Proc. Natl. Acad. Sci. U S A*. **116**, 10705–10710 (2019). 31061125 10.1073/pnas.1820210116 PMC6561172

[60] Tornberg, A. & Vandkilde, H. Modelling age at death reveals nordic corded ware paleodemography. *Archaeol Anthropol. Sci* **17**, 38 (2025).

[61] Alt, K. W. et al. A massacre of early neolithic farmers in the high Pyrenees at Els trocs, Spain. *Sci. Rep.* **10**, 2131 (2020). 32034181 10.1038/s41598-020-58483-9 PMC7005801

[62] Sanday, P. R. *Divine Hunger: Cannibalism as a Cultural System* (Cambridge University Press, 1986).

[63] Villa, P. et al. Un Cas de cannibalisme Au néolithique. *Gallia Préhistoire* **29**, 143–171 (1986).

[64] Fibiger, L., Ahlström, T., Meyer, C. & Smith, M. Conflict, violence, and warfare among early farmers in Northwestern Europe. *Proc. Natl. Acad. Sci. U. S. A.* 120, e2209481119 (2023). 10.1073/pnas.2209481119 PMC9942812 36649427

[65] Schulting, R. J. & Fibiger, L. *Sticks, Stones, and Broken Bones: Neolithic Violence in a European Perspective*OUP Oxford,. (2012).

[66] Schulting, R. J. & Wysocki, M. ‘in this chambered tumulus were found cleft skulls… An assessment of the evidence for cranial trauma in the British Neolithic. *Proc. Prehist. Soc.* 71, 107–138 (2005).

[67] Smith, M. J. Routledge,. The war to begin all wars? Contextualizing violence in Neolithic Britain. in *The Routledge Handbook of the Bioarchaeology of Human Conflict* (eds. Knüsel, C. & Smith, M. J.) 109–126 (2013).

[68] Molloy, B. & Horn, C. Weapons, warriors and warfare in bronze age Europe. in *The Cambridge World History of Violence* 117–141Cambridge University Press, (2020).

[69] Moreno-Ibáñez, M. Á., Saladié, P., Morales, J. I., Cebrià, A. & Fullola, J. M. Inhumation and cremation: identifying funerary practices and reuse of space through forensic taphonomy at Cova Foradada (Calafell, Spain). *Archaeol Anthropol. Sci* **14**, 57 (2022).

[70] Mercadal, O. La Costa de can Martorell (Dosrius, El maresme, Barcelona): Muerte y violencia en una comunidad del litoral catalán durante el tercer milenio cal. BC. In (ed. Canyellas, A) *III Congreso Del Neolítico En La Península Ibérica* 671–680 (Dosrius, El Maresme, 2005).

[71] Fernández-Crespo, T. et al. Large-scale violence in late neolithic Western Europe based on expanded skeletal evidence from San Juan ante Portam Latinam. *Sci. Rep.* **13**, 17103 (2023). 37919365 10.1038/s41598-023-43026-9 PMC10622514

[72] AlQahtani, S. J., Hector, M. P. & Liversidge, H. M. Brief communication: the London atlas of human tooth development and eruption. *Am. J. Phys. Anthropol.* **142**, 481–490 (2010). 20310064 10.1002/ajpa.21258

[73] Schaefer, M. C., Scheuer, L. & Black, S. *Juvenile Osteology: A Laboratory and Field Manual* (Academic, 2014).

[74] Potts, R. B. & Shipman, P. Cutmarks made by stone tools on bones from Olduvai gorge, Tanzania. *Nature* **291**, 577–580 (1981).

[75] Domínguez-Solera, S. D. & Domínguez-Rodrigo, M. A taphonomic study of bone modification and of tooth-mark patterns on long limb bone portions by suids. *International Journal of Osteoarchaeology* vol. 19 345–363 Preprint at (2009). 10.1002/oa.987

[76] Lyman, R. L. *Quantitative Paleozoology* (Cambridge University Press, 2008).

[77] Blumenschine, R. J. & Selvaggio, M. M. Percussion marks on bone surfaces as a new diagnostic of hominid behavior. *Nature* **333**, 763–765 (1988).

[78] Villa, P. & Mahieu, E. Breakage patterns of human long bones. *J. Hum. Evol.* **21**, 27–48 (1991).

[79] Téllez, E. et al. Incidental burning on bones by neanderthals: the role of fire in the Qa level of abric Romaní rock-shelter (Spain). *Archaeol. Anthropol. Sci.* **14**, 119 (2022).

[80] Bronk Ramsey, C., Schulting, R. J., Bazaliiskii, V. I., Goriunova, O. I. & Weber, A. W. Spatio-temporal patterns of cemetery use among middle holocene hunter-gatherers of Cis-Baikal, Eastern Siberia. *Archaeol. Res. Asia*. **25**, 100253 (2021).

[81] Reimer, P. J. et al. The IntCal20 Northern hemisphere radiocarbon age calibration curve (0–55 cal kBP). *Radiocarbon* **62**, 725–757 (2020).

[82] Pourmand, A., Prospero, J. M. & Sharifi, A. Geochemical fingerprinting of trans-Atlantic African dust based on radiogenic Sr-Nd-Hf isotopes and rare Earth element anomalies. *Geology* **42**, 675–678 (2014).

[83] Pourmand, A. & Dauphas, N. Distribution coefficients of 60 elements on TODGA resin: application to ca, lu, hf, U and Th isotope geochemistry. *Talanta* **81**, 741–753 (2010). 20298848 10.1016/j.talanta.2010.01.008

