# Supplementary material for: Evidence of neolithic cannibalism among farming communities at El Mirador cave, Sierra de Atapuerca, Spain
Source: Sci Rep. 2025 Aug 7;15:26648. doi: 10.1038/s41598-025-10266-w (PMC12331932; doi:10.1038/s41598-025-10266-w)
Supplement: Supplementary file 1 — Supplementary Material 1 [file 41598_2025_10266_MOESM1_ESM.docx]

*Supplementary Materials for:*

**Evidence of Neolithic cannibalism among farming communities at El Mirador cave (Sierra de Atapuerca, Spain)**

Palmira Saladié*^1,2,3^, Francesc Marginedas*^,1,2^, Juan Ignacio Morales^1,2^, Josep María Vergès^1, 2^, Ethel Allué^1,2^, Isabel Expósito^1,2^, Marina Lozano^1,2^, Patricia Martín^1,2^, Javier Iglesias-Bexiga^4^, Marta Fontanals^2,1^, Roser Marsal^5^, Raquel Hernando^6^, Aitor Burguet-Coca^1,2,7^, Antonio Rodríguez-Hidalgo*^8,1^

*Corresponding Authors (PS psaladie@iphes.com; FM francescm63@gmail.com; ARH antonio.rodriguez@iam.csic.es)

1 Catalan Institute of Human Paleoecology and Social Evolution (IPHES), Zona Educacional 4, Campus Sescelades URV (Building W3), 43007, Tarragona, Spain.

2 Area of Prehistory, Rovira i Virgili University, Campus Catalunya. Avinguda de Catalunya 35, 43003, Tarragona, Spain.

3 Unit Associated with CSIC, Department of Paleobiology, National Museum of Natural Sciences (CSIC), Calle José Gutiérrez Abascal, 2, 28006, Madrid, Spain.

4 Department of Biology, Autonomous University of Madrid (UAM). C/ Francisco Tomás y Valiente 1 Campus de Cantoblanco, UAM, 28-049 Madrid, Spain.

5 Water Research Institute (IdRA-UB), Faculty of Chemistry, University of Barcelona, C/ Martí Franquès 1-11, 08028, Barcelona, Spain.6. National Center for Research on Human Evolution (CENIEH), Paseo Sierra de Atapuerca, 3, 09002, Burgos, Spain.

6 Spanish National Research Council, Institute of Archaeology-Mérida (CSIC-Junta de Extremadura), Plaza de España 15, 06800, Mérida, Spain.

7 Leiden University, Faculty of Archaeology, Department of Archaeological Sciences, Einsteinweg 2, 2333CC, Leiden, The Netherlands.

# Supplementary Notes

## Supplementary Note 1: El Mirador cave

El Mirador cave (Burgos, Spain) is part of a karst system located on the southern slope of the Sierra de Atapuerca at 1,033 meters above sea level (Figure S1). Its entrance measures approximately 23 meters wide by 4 meters high and the cave extends about 15 meters deep (Figure S2). Excavations at the site began in 1999 and continue to this day. During the first 10 years, excavations focused on a 6-square-meter area in the central part of the surface, from now on referred to in this paper as the ‘Test Pit’ (Figure S3). The El Mirador cave excavation team chose the location of the Test Pit based on data provided by an electrical resistivity tomography test, which indicated that the area did not contain large blocks that would hinder the continuity of the excavation [**^1^**.](https://paperpile.com/c/x4U1di/qPhR) The excavation reached a depth of 20 meters, within which 14 meters of Pleistocene sediments and 6 meters of Holocene sediments were documented. Of these, 4 meters correspond to the stratified *fumier* Neolithic levels MIR24 to MIR6 associated with livestock activities  [**^1^**](https://paperpile.com/c/x4U1di/qPhR) with an unmodelled age dated at 5593–5331 cal BP (Table S1). The remaining part of the Holocene sequence comprises Middle Bronze Age

occupations, beginning at 3811–3487 cal BP (MIR4 and MIR3A) (Table S1, S2). In the Test Pit, Neolithic and Bronze Age occupations are separated by MIR5, an archaeologically sterile level only a few centimeters thick[**^1^**.](https://paperpile.com/c/x4U1di/qPhR) Essentially, the Holocene sequence reveals that the primary use of El Mirador cave was as a sheepfold[**^2^**.](https://paperpile.com/c/x4U1di/829U)

During the excavation season in 2009, interventions began in two new sectors, named 100 and 200 (S100 and S200 from here on), which are in contact with the current cave wall (Figure S3). The location of these new sectors was chosen based on the geometry of the Holocene and Pleistocene deposits, indicating gravitational sediment deformation possibly related to the existence of another gallery [**^1^**.](https://paperpile.com/c/x4U1di/qPhR) Both sectors were excavated in a stepped manner following the roof of the cave, allowing the roofline to be traced and documented as it retreated into the interior and the stratigraphic variations in different areas to be noted. In S200, a small natural chamber was uncovered that was used for a collective funerary deposition during the Chalcolithic period (level MIR203) (Figure S4). The unmodelled ^14^C results from the dating of different individuals provides a maximum time span of 5214–4862 to 4786–4421 cal BP. A 3-meter Bronze Age sequence (levels MIR106–MIR103) was identified in S100.

Most of these levels correspond to the cave’s use as a domestic livestock enclosure.

However, level MIR106 corresponds to an individual burial dated at 3824–3575 cal BP (Figure S5). Subsequent levels (MIR 107–109) complete the testimony of the continuity of the use of the site for livestock activities during the Neolithic[**^1,3^**.](https://paperpile.com/c/x4U1di/qPhR+HcuX)

The levels corresponding to the Bronze Age and Neolithic livestock enclosures are primarily composed of animal excrement, characterized by alternating layers of burnt and unburnt dung. The burnt levels, often with lenticular morphology, are the result of the practice of piling up corral waste and burning it to reduce its volume and eliminate parasites. Thus, the stratigraphic sequence of El Mirador cave is composed of burned and unburned layers of dung[**^1,4^**.](https://paperpile.com/c/x4U1di/qPhR+9zlJ) Other refuse from domestic activities (fauna, ceramics, lithic and bone industry, seeds, etc.) and activities related to animal husbandry are mixed in with the sediments[**^5^**.](https://paperpile.com/c/x4U1di/DbUU) The sequence of Holocene burning events at El Mirador cave underwent various archaeomagnetic analyses related to magnetic susceptibility anisotropy[**^6,7^**.](https://paperpile.com/c/x4U1di/V5N7+IFDA) The results determined the absence of post-depositional movements of archaeological sediments

## Supplementary Note 2: The MNI and Age

The repetition of the diaphysis of the right femurs allowed the establishment of a Minimum Number of Individuals (MNI) of nine in the set of remains with human-induced modifications, from sectors 100 and 200 of La Cueva de El Mirador. Among them, it can be seen that at least three had small dimensions. However, the absence of epiphyses did not allow the age at death of the nine individuals to be determined. For this purpose, other elements were used, and the age of skull fusion as well as the eruption and wear patterns of teeth were considered (Table S5)**^8^**.

In the assemblage, there are three occipital fragments, two of which refit. Both preserve the occipital squama, in which the pars lateralis and pars basilaris are unfused, allowing the identification of two individuals (Individuals 1 and 2) who were less than 5–7 years old at the time of death.

The right hemimandible ATA16-MIR202-N37-58 had the Dm2 and M1 lost post-mortem, and M2 and M3 germs present inside the alveoli, as well as alveoli for I1, I2 (right and left), C, P1, and M1 (right). The Dm2 shows heavy wear on all cusps, and the M2 and M3 are in the crypts. The M1 is missing, but all alveoli are open, suggesting that this tooth had erupted and was lost post-mortem. The age of this individual was estimated to be around 6–10 years.

Individual 4 was identified through the hemimandible ATA16-MIR102-S13-20. It is a left hemimandible with the chin, canine, M1, M2, and M3. The M1 exhibits wear on the anterior buccal cusp. The M2 shows no signs of wear, and the M3 remains in the crypt. Additionally, the alveoli of I1, I2 (right and left), and left P1 and P2 are preserved. This individual was estimated to be 15–17 years old at the time of death.

The next individual is represented by the left hemimandible ATA16-MIR102-T12-21, which has M3 in the crypts with an incompletely formed crown, and alveoli for I1, I2 (right and left), and left C, P1, P2, M1, and M2. This individual was estimated to be 12–15 years old, slightly younger than Individual 4.

Individual 6 was identified through a left maxilla with M1, M2, and M3 present, and the alveolus of PM2. M1 shows wear on all four cusps; M2 exhibits wear on the two anterior cusps and the posterior lingual cusp, with minimal wear on the vestibular cusp. This individual was estimated to be between 20–25 years old at the time of death.

Individual 7 is the last for whom age at death could be determined. This individual was identified by a hemimandible, ATA16-MIR102-T12-6, without teeth, although the roots of the M2 are visible. Significant bone resorption is also observed in this specimen.

# Supplementary Figures

*Supplementary Fig. 1. Aerial image of El Mirador cave entrance. El Mirador cave images*


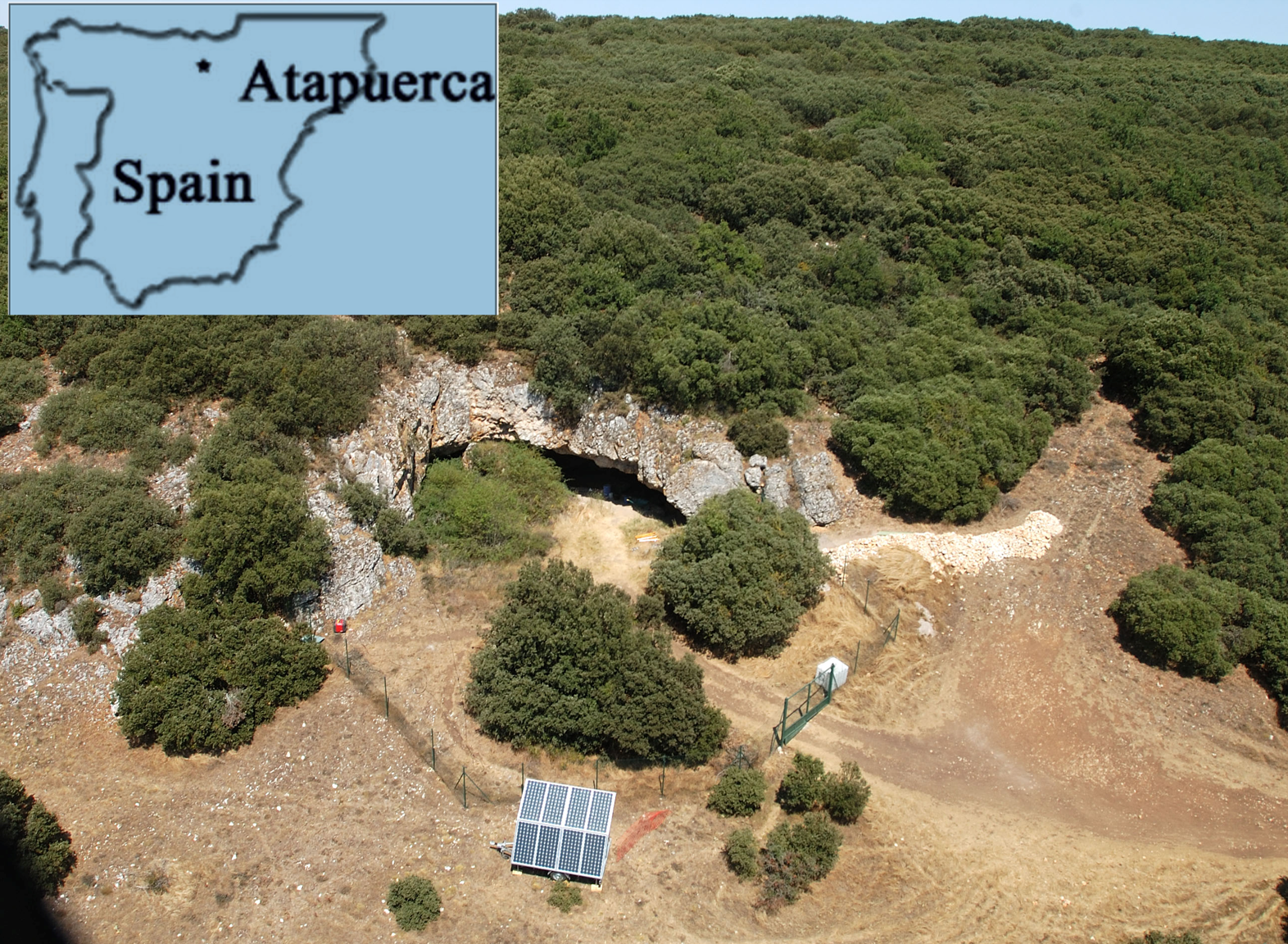


*Supplementary Fig. 2. General view of the interior of the cave. Photography IPHES*


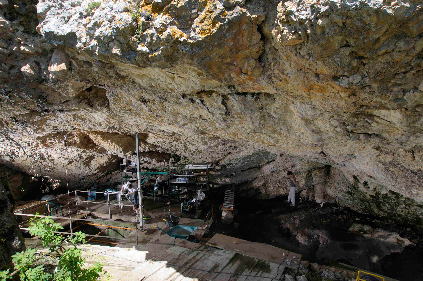


*Supplementary Fig. 3. D scan images of El Mirador cave showing the location of the human remains in the test pit, S100, and S200. The blue circle indicates the location of the cannibalized remains in the test pit. The orange oval indicates the location of the individual inhumation. The red dots (not in anatomical connection) and green dots (in anatomical connection) indicate the location of the skulls in S200 (from ^20^). Photography IPHES*


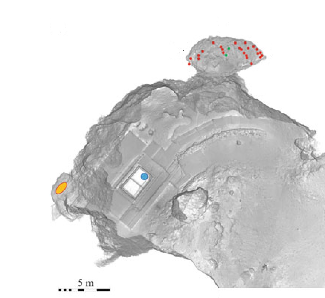


*Supplementary Fig. 4. Accumulation of cannibalized remains in MIR4. Photography IPHES*


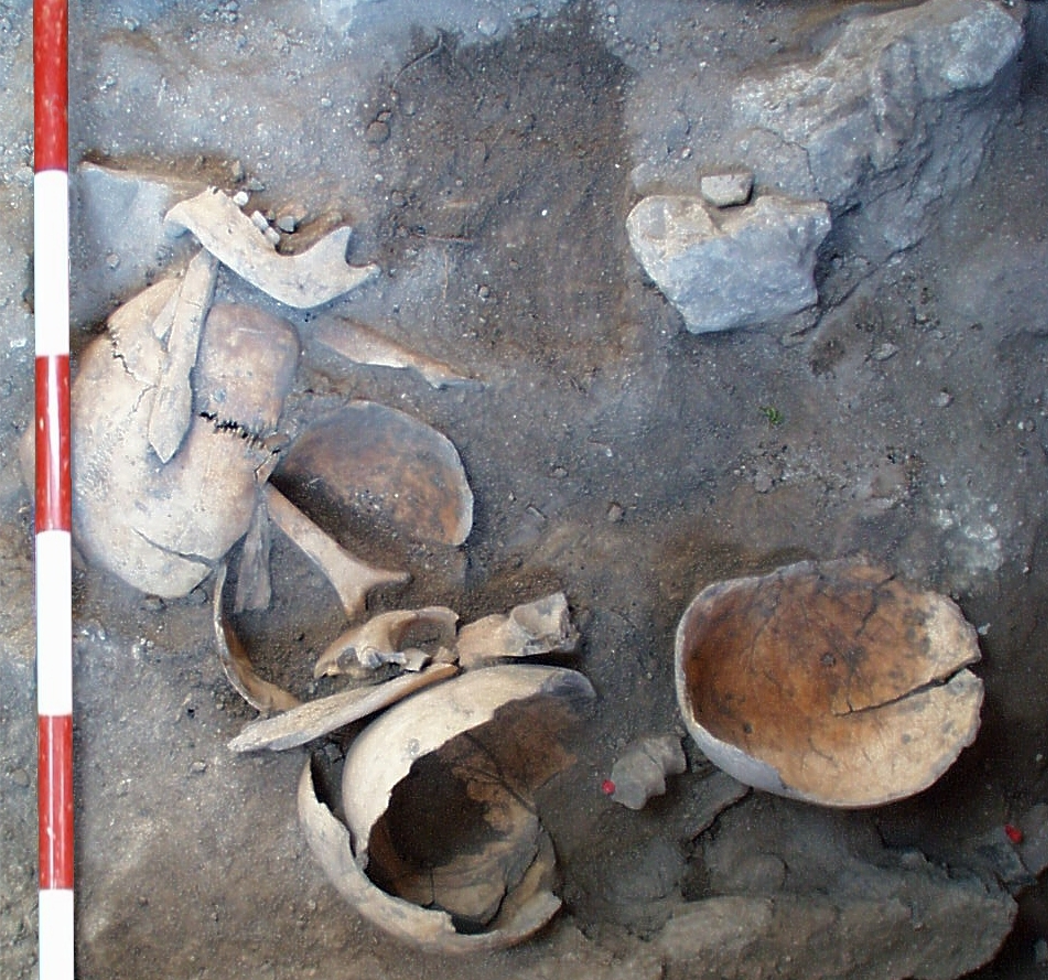


*Supplementary Fig. 5. Individual funerary deposit of MIR 106. Photography IPHES*


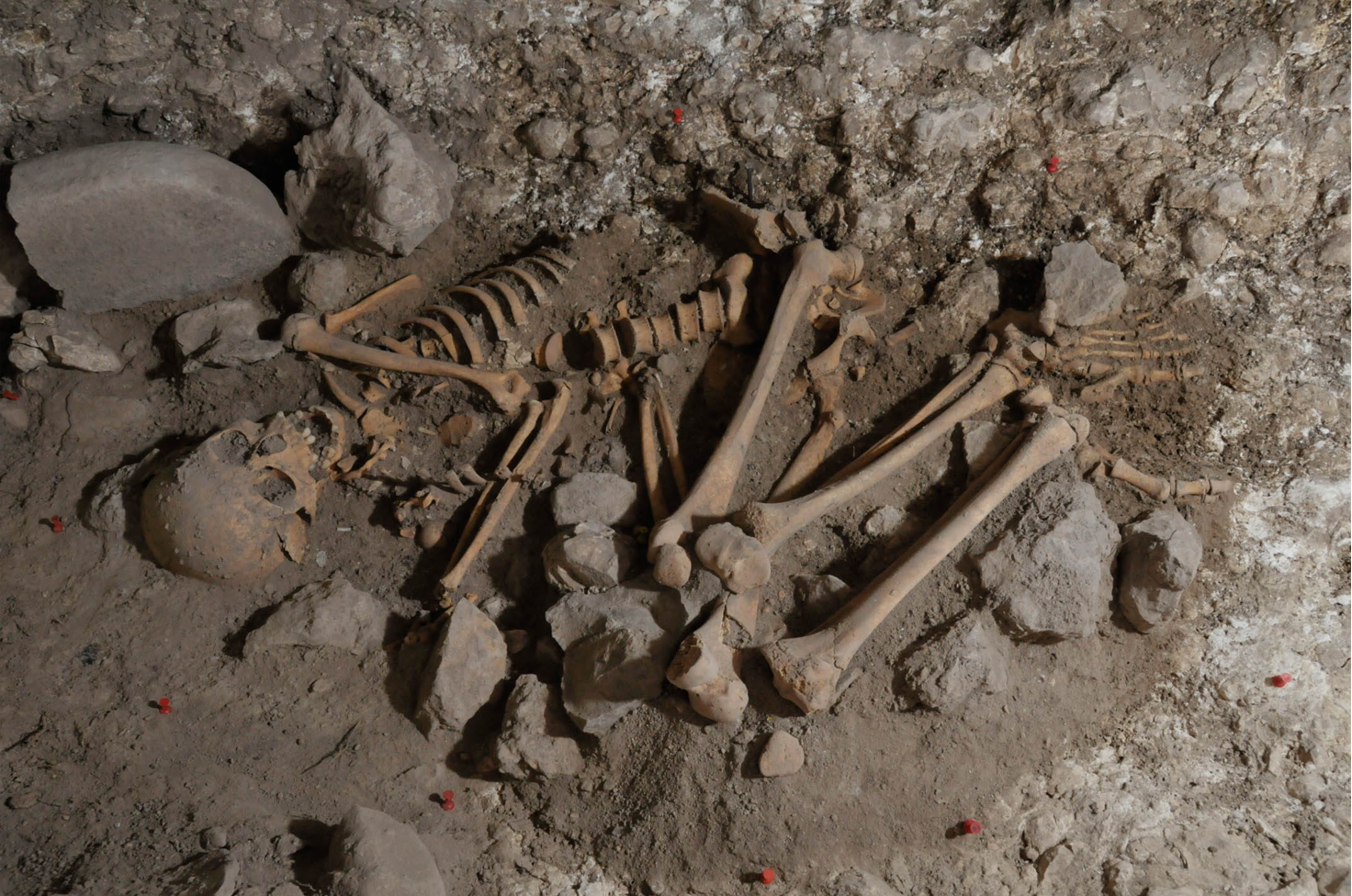


*Supplementary Fig. 6. Surface image with remains of the collective burial in S200. Photography IPHES*


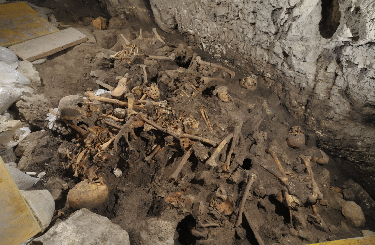


*Supplementary Fig. 7. Fragments of humeri directly dated.* *Photography M.D.Guillen/IPHES*


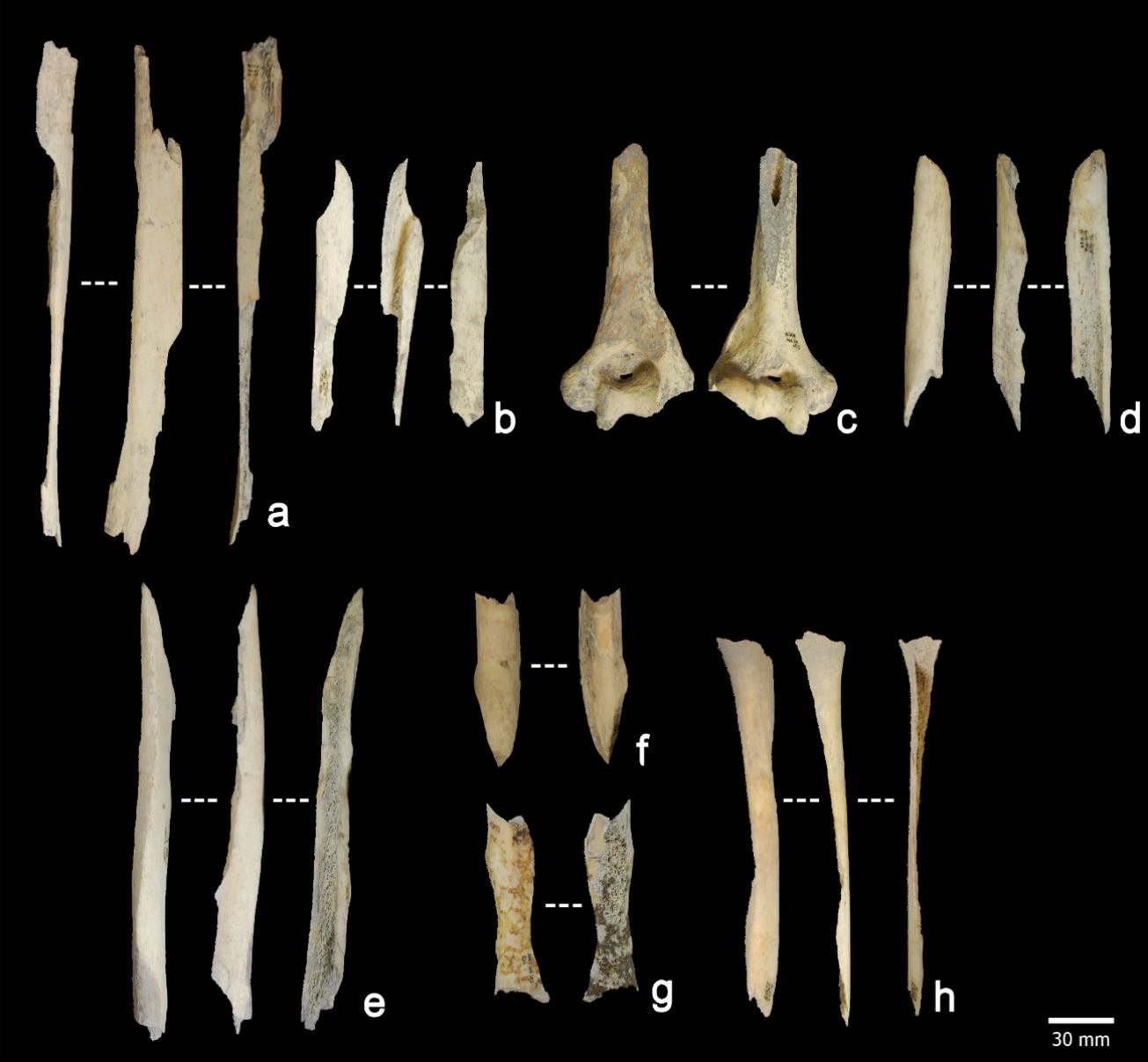


(a*) ATA11-MIR202-S36-26, (b) ATA13-MIR202-P39-9, (c) ATA14-MIR2,02-T33-*

*92, (d) ATA14-MIR202-O37-86, (e) ATA16-MIR202-T35-54, (f) ATA16-MIR204O38-6, (g) ATA17-MIR204-N38-15, (h) ATA16-MIR102-T13-91, (i) ATA15MIR204-Q37-11ATA16-MIR204-O38-6.*

*Supplementary Fig.8. Fragments of femurs with which the Minimum Number of Individuals of the assemblage has been established. Photography M.D.Guillen/IPHES*


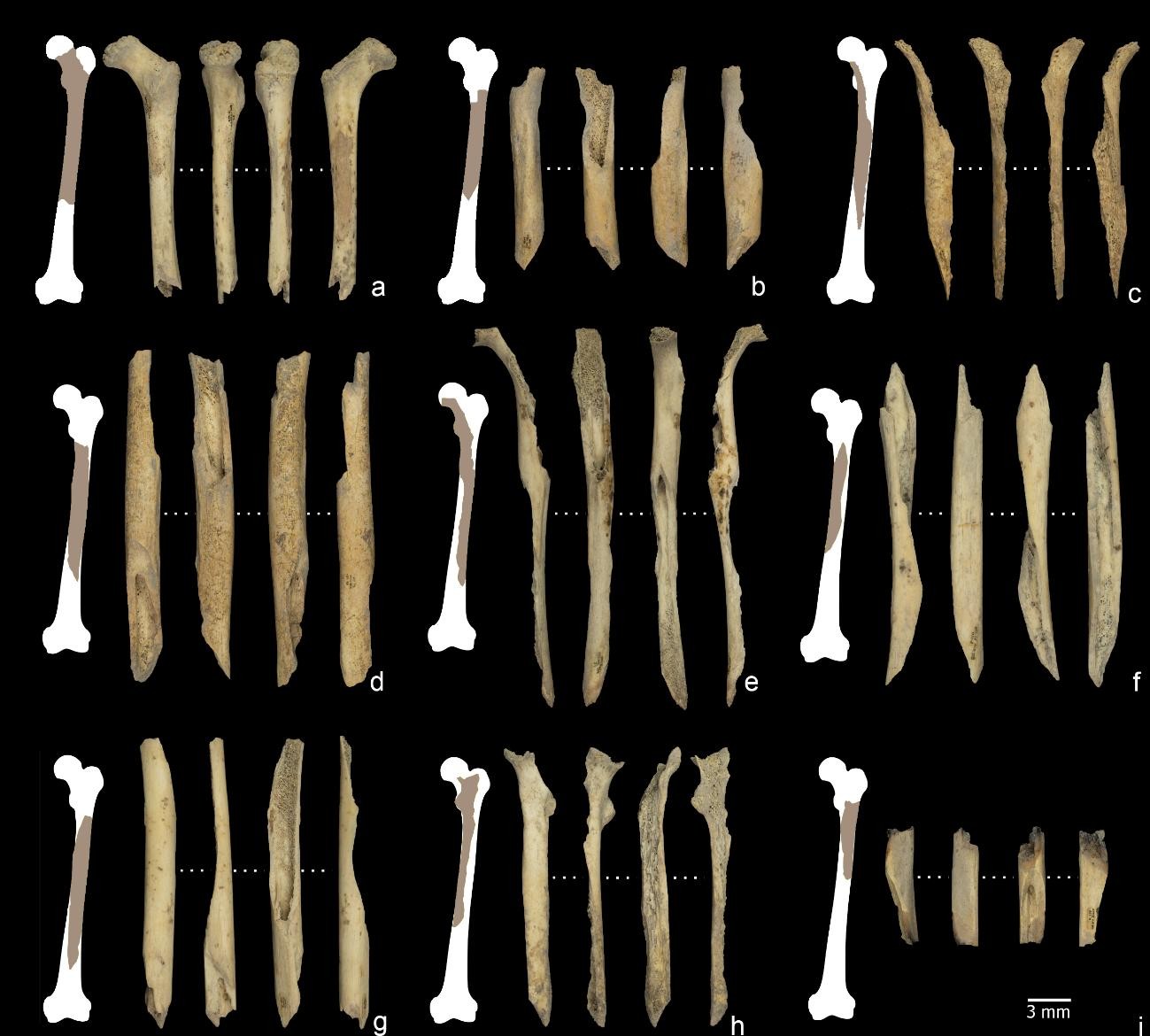


(a*) ATA11-MIR202-S36-26, (b) ATA13-MIR202-P39-9, (c) ATA14-MIR2,02-T33-*

*92, (d) ATA14-MIR202-O37-86, (e) ATA16-MIR202-T35-54, (f) ATA16-MIR204O38-6, (g) ATA17-MIR204-N38-15, (h) ATA16-MIR102-T13-91, (i) ATA15MIR204-Q37-11ATA16-MIR204-O38-6.*

*Supplementary Fig.9. Confocal microscope images of cut marks El Mirador cave. Photography F.Marginedas/IPHES*

.  
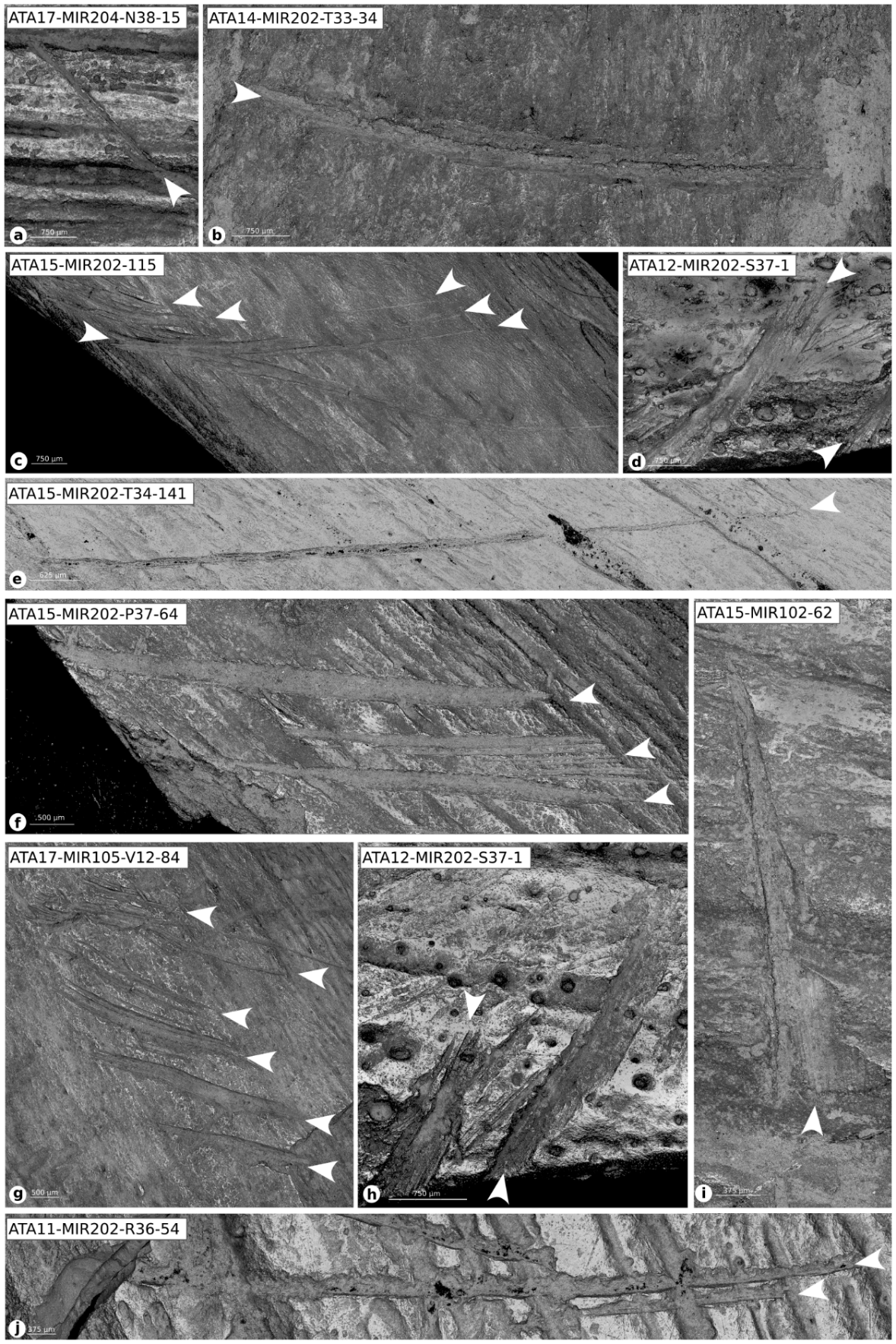


*Example defleshing cut marks (white arrow) on a femur (a, i), rib (b, c), tibia (d, e, g, h, j) and humerus (f). Scale bars are shown at the bottom of each image. Specimen numbers are indicated in white boxes*

# Supplementary Tables

*Table S1. Radiometric dating of the Pleistocene and Holocene levels from El Mirador cave.*

| **Level** | **Group** | **Phase** | **Sample** | **Lab number** | **C14** | **SD** | **CalBP (95%)** | | **CalBC (95%)** | | **d13C o/oo** | **Reference** |
| --- | --- | --- | --- | --- | --- | --- | --- | --- | --- | --- | --- | --- |
| MIR 51/3-loess | Loess | 1 | Pollen | Beta-220915 | 12480 | 40 | 14985 | 14345 | -13036 | -12396 | 27,20 | [2](https://paperpile.com/c/x4U1di/829U) |
| MIR 51/2 | Upper Palaeolithic | 2 | Charcoal | Beta-208136 | 11610 | 40 | 13583 | 13353 | -11634 | -11404 | 25,00 | [2](https://paperpile.com/c/x4U1di/829U) |
| MIR 51/2 | Upper Palaeolithic | 2 | Charcoal | Beta-208135 | 11470 | 40 | 13454 | 13242 | -11505 | -11293 | 24,00 | [2](https://paperpile.com/c/x4U1di/829U) |
| MIR 49/01 | Rock fall | 3 | Small mammals | Beta-631289 | 9900 | 30 | 11396 | 11236 | -9447 | -9287 |  | [1](https://paperpile.com/c/x4U1di/qPhR) |
| MIR 49/02 | Rock fall | 3 | Small mammals | Beta-631290 | 7740 | 30 | 8590 | 8430 | -6641 | -6481 |  | [1](https://paperpile.com/c/x4U1di/qPhR) |
| MIR 24 | Neolithic | 4 | Charcoal | Beta-197386 | 7060 | 40 | 7969 | 7791 | -6020 | -5842 | 22,90 | [2](https://paperpile.com/c/x4U1di/829U) |
| MIR 21 | Neolithic | 4 | Charcoal | Beta-197385 | 6380 | 40 | 7423 | 7172 | -5474 | -5223 | 22,90 | [2](https://paperpile.com/c/x4U1di/829U) |
| MIR 23 | Neolithic | 4 | Charcoal | Beta-208134 | 6320 | 50 | 7420 | 7079 | -5471 | -5130 | 23,(80) | [2](https://paperpile.com/c/x4U1di/829U) |
| MIR 22 | Neolithic | 4 | Charcoal | Beta-208133 | 6150 | 40 | 7164 | 6909 | -5215 | -4960 | 22,30 | [2](https://paperpile.com/c/x4U1di/829U) |
| MIR 19 | Neolithic | 4 | Charcoal | Beta-182040 | 6130 | 50 | 7164 | 6884 | -5215 | -4935 | 24,70 | [2](https://paperpile.com/c/x4U1di/829U) |
| MIR 18 | Neolithic | 4 | Charcoal | Beta-208132 | 6120 | 40 | 7158 | 6893 | -5209 | -4944 | 23,00 | [2](https://paperpile.com/c/x4U1di/829U) |
| MIR 24 | Neolithic | 4 | Charcoal | Beta-220914 | 6110 | 40 | 7159 | 6884 | -5210 | -4935 | 23,40 | [2](https://paperpile.com/c/x4U1di/829U) |
| MIR 20 | Neolithic | 4 | Charcoal | Beta-197384 | 6100 | 50 | 7158 | 6(80)1 | -5209 | -4852 | 22,90 | [2](https://paperpile.com/c/x4U1di/829U) |
| MIR 109 | Neolithic | 4 | Fauna bone | Beta-510343 | 5890 | 30 | 6787 | 6652 | -4838 | -4703 | 20,90 | [1](https://paperpile.com/c/x4U1di/qPhR) |
| MIR 16 | Neolithic | 4 | Charcoal | Beta-181088 | 5700 | 70 | 6657 | 6316 | -4708 | -4367 | 25,00 | [2](https://paperpile.com/c/x4U1di/829U) |
| MIR 107 | Neolithic | 4 | Charcoal | Beta-510341 | 5640 | 30 | 6491 | 6316 | -4542 | -4367 | 21,50 | [1](https://paperpile.com/c/x4U1di/qPhR) |
| MIR 108 | Neolithic | 4 | Fauna bone | Beta-510342 | 5610 | 30 | 64(80) | 6305 | -4531 | -4356 | 20,40 | [1](https://paperpile.com/c/x4U1di/qPhR) |
| MIR 14 | Neolithic | 4 | Charcoal | Beta-220913 | 5480 | 40 | 6392 | 6198 | -4443 | -4249 | 24,30 | [2](https://paperpile.com/c/x4U1di/829U) |
| MIR 13 | Neolithic | 4 | Charcoal | Beta-208131 | 5470 | 40 | 6391 | 6192 | -4442 | -4243 | 21,(80) | [2](https://paperpile.com/c/x4U1di/829U) |
| MIR 11 | Neolithic | 4 | Charcoal | Beta-181087 | 5360 | 50 | 62(80) | 6000 | -4331 | -4051 | 23,90 | [2](https://paperpile.com/c/x4U1di/829U) |
| MIR 9 | Neolithic | 4 | Charcoal | Beta-220912 | 5090 | 40 | 5921 | 5739 | -3972 | -3790 | 22,60 | [2](https://paperpile.com/c/x4U1di/829U) |
| MIR 205 | Neolithic | 4 | Fauna bone | Beta-510345 | 4970 | 30 | 5844 | 5600 | -3895 | -3651 | 20,00 | [1](https://paperpile.com/c/x4U1di/qPhR) |
| MIR 8 | Neolithic | 4 | Charcoal | Beta-181086 | 4970 | 40 | 5882 | 5596 | -3933 | -3647 | 23,60 | [2](https://paperpile.com/c/x4U1di/829U) |
| MIR 6 | Neolithic | 4 | Charcoal | Beta-153367 | 4780 | 40 | 5593 | 5331 | -3644 | -3382 | 23,50 | [14](https://paperpile.com/c/x4U1di/D1Rz) |
| MIR 101 | Cannibalized scattered | 5 | Human bone | Beta-587098 | 4980 | 30 | 5860 | 5601 | -3911 | -3652 | -18,40 |  |

| MIR 206 | Cannibalized scattered | 5 | Human bone | Beta-587097 | 4970 | 30 | 5844 | 5600 | -3895 | -3651 | -19,30 |  |
| --- | --- | --- | --- | --- | --- | --- | --- | --- | --- | --- | --- | --- |
| MIR 204 | Cannibalized scattered | 5 | Human bone | Beta-587095 | 4940 | 30 | 5728 | 5596 | -3779 | -3647 | -18,90 |  |
| MIR 204 | Cannibalized scattered | 5 | Human bone | Beta-510344 | 4930 | 30 | 5720 | 5594 | -3771 | -3645 | 18,90 | [1](https://paperpile.com/c/x4U1di/qPhR) |
| MIR 205 | Cannibalized scattered | 5 | Human bone | Beta-587096 | 4910 | 30 | 5715 | 5587 | -3766 | -3638 | -19,10 |  |
| MIR 203 | Cannibalized scattered | 5 | Human bone | Beta-587094 | 4900 | 30 | 5714 | 5585 | -3765 | -3636 | -18,90 |  |
| MIR 105 | Cannibalized scattered | 5 | Human bone | Beta-587099 | 4880 | 30 | 5705 | 5488 | -3756 | -3539 | -19,50 |  |
| MIR 201 | Cannibalized scattered | 5 | Human bone | Beta-587093 | 4880 | 30 | 5705 | 5488 | -3756 | -3539 | -19,10 |  |
| MIR 206 | Collective burial | 6 | Human bone | Beta-514855 | 4400 | 30 | 5214 | 4862 | -3265 | -2913 | 20,00 |  |
| MIR203 | Collective burial | 6 | Human bone | Beta-416455 | 4320 | 30 | 4962 | 4838 | -3013 | -2889 |  |  |
| MIR 203 | Collective burial | 6 | Human bone | Beta-296227 | 4220 | 30 | 4854 | 4627 | -2905 | -2678 | 18,70 | [2](https://paperpile.com/c/x4U1di/829U) |
| MIR201 | Collective burial | 6 | Human bone | Beta-416458 | 4210 | 30 | 4849 | 4624 | -2900 | -2675 |  |  |
| MIR201 | Collective burial | 6 | Human bone | Beta-416456 | 4170 | 30 | 4832 | 4581 | -2883 | -2632 |  |  |
| MIR 203 | Collective burial | 6 | Human bone | Beta-296225 | 4100 | 30 | 4813 | 4449 | -2864 | -2500 | 18,90 | [2](https://paperpile.com/c/x4U1di/829U) |
| MIR201 | Collective burial | 6 | Human bone | Beta-416457 | 4050 | 30 | 4786 | 4421 | -2837 | -2472 |  |  |
| MIR 5 | Hiatus | 7 | Pellet | Beta-521985 | 4010 | 30 | 4567 | 4414 | -2618 | -2465 | -19,10 | [15](https://paperpile.com/c/x4U1di/OSnc) |
| MIR 4 (pit) | Cannibalized pit | 8 | Human bone | Beta-182041 | 3900 | 40 | 4423 | 4158 | -2474 | -2209 | 19,20 | [16](https://paperpile.com/c/x4U1di/nE3G) |
| MIR 4 (pit) | Cannibalized pit | 8 | Human bone | Beta-182042 | 3830 | 40 | 4405 | 4095 | -2456 | -2146 | 18,(80) | [16](https://paperpile.com/c/x4U1di/nE3G) |
| MIR 4 (pit) | Cannibalized pit | 8 | Human bone | Beta-153365 | 3670 | 40 | 4145 | 3886 | -2196 | -1937 | 19,30 | [14](https://paperpile.com/c/x4U1di/D1Rz) |
| MIR 106 | Individual Inhumation | 9 | Human bone | Beta-296226 | 3430 | 30 | 3824 | 3575 | -1875 | -1626 | 19,40 | [14](https://paperpile.com/c/x4U1di/D1Rz) |
| MIR 4 (bottom) | Bronze Age | 10 | Charcoal | Beta-153366 | 3380 | 40 | 3811 | 3487 | -1862 | -1538 | 23,(80) | [14](https://paperpile.com/c/x4U1di/D1Rz) |
| MIR 104 | Bronze Age | 10 | Charcoal | Beta-339095 | 3350 | 30 | 3685 | 3485 | -1736 | -1536 | 22,40 | [2](https://paperpile.com/c/x4U1di/829U) |
| MIR 103 | Bronze Age | 10 | Charcoal | Beta-339094 | 3190 | 30 | 3456 | 3364 | -1507 | -1415 | 22,(80) | [2](https://paperpile.com/c/x4U1di/829U) |
| MIR 4 (top) | Bronze Age | 10 | Charcoal | Beta-154894 | 3040 | 40 | 3363 | 3082 | -1414 | -1133 | 23,90 | [15](https://paperpile.com/c/x4U1di/OSnc) |

*Table S2. Results of the Bayesian model for the Holocene sequence at El Mirador cave as calculated using OxCal v4.4.*

**from to % from to % Acomb A L P C**

| R_Date 10-Beta-154894 | 3363 | 3082 95.45 | 3363 | 3082 | 95.45 |  | 99.9 |  |  | 99.9 |
| --- | --- | --- | --- | --- | --- | --- | --- | --- | --- | --- |
| R_Date 10-Beta-339094 | 3456 | 3364 95.45 | 3456 | 3364 | 95.45 |  | 99.7 |  |  | 99.9 |
| R_Date 10-Beta-339095 | 3685 | 3485 95.45 | 3641 | 3481 | 95.45 |  | 102.7 |  |  | 100 |
| R_Date 10-Beta-153366 | 3811 | 3487 95.45 | 3682 | 3484 | 95.45 |  | 97.3 |  |  | 99.9 |
| **Boundary Start Bronze Age** |  |  | **3795** | **3555** | **95,45** |  |  |  |  | **99,9** |

| **Boundary End Individual burial** |  | **3815** | **95,45** |  |  |  |  | **99,8** |
| --- | --- | --- | --- | --- | --- | --- | --- | --- |
| *Interval Individual Burial* |  | 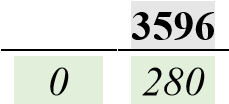 | *95,45* |  |  |  |  | *99,9* |
| R_Date 9-Beta-296226 | 3813 3638 95.45 | 3819 3642 | 95.45 |  | 65.5 |  |  | 99.7 |
| **Boundary Start Individual burial** |  | **3966 3645** | **95,45** |  |  |  |  | **99,8** |

| **Boundary End Cannibal Event 2** |  |  | **95,45** |  |  |  |  | **99,9** |
| --- | --- | --- | --- | --- | --- | --- | --- | --- |
| *Interval Cannibal Event 2* |  | 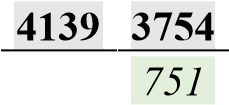*126* | *95,45* |  |  |  |  | *99,9* |
| R_Date 8-Beta-153365 | 4145 3886 95.45 | 4151 3913 | 95.45 |  | 95.4 |  |  | 99.9 |
| R_Date 8-Beta-182042 | 4405 4095 95.45 | 4390 4093 | 95.45 |  | 105 |  |  | 99.9 |
| R_Date 8-Beta-182041 | 4423 4158 95.45 | 4414 4155 | 95.45 |  | 88.5 |  |  | 99.9 |
| **Boundary Start Cannibal Event 2** |  | **4604 4184** | **95.45** |  |  |  |  | **99.9** |

| **Boundary End Collective Burial** |  | | **4781** | **95,45** |  |  |  |  | **99,6** |
| --- | --- | --- | --- | --- | --- | --- | --- | --- | --- |
| *Interval Collective Burial* |  |  | 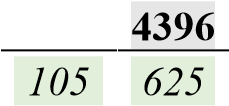 | *95,45* |  |  |  |  | *99,7* |
| R_Date 6-Beta-416457 | 4786 | 4421 95.45 | 4807 4447 | 95.45 |  | 54.5 |  |  | 99.7 |
| R_Date 6-Beta-296225 | 4813 | 4449 95.45 | 4818 4525 | 95.45 |  | 96.8 |  |  | 99.9 |
| R_Date 6-Beta-416456 | 4832 | 4581 95.45 | 4835 4615 | 95.45 |  | 101.2 |  |  | 99.9 |
| R_Date 6-Beta-416458 | 4849 | 4624 95.45 | 4851 4628 | 95.45 |  | 103.1 |  |  | 99.9 |
| R_Date 6-Beta-296227 | 4854 | 4627 95.45 | 4855 4646 | 95.45 |  | 103.9 |  |  | 99.9 |
| R_Date 6-Beta-416455 | 4962 | 4838 95.45 | 4955 4836 | 95.45 |  | 113.9 |  |  | 100 |
| R_Date 6-Beta-514855 | 5214 | 4862 95.45 | 5017 4855 | 95.45 |  | 101.1 |  |  | 99.9 |
| **Boundary Start Collective Burial** |  |  | **5081 4864** | **95.45** |  |  |  |  | **99.9** |

| **Boundary End Cannibal Event 1** | **5650** | **95.45** |  |  |  |  | **50.4** |
| --- | --- | --- | --- | --- | --- | --- | --- |
| *Interval Cannibal Event 1* | 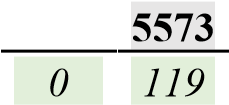 | *95,45* |  |  |  |  | *49,2* |
| R_Date 5-Beta-587093 | 5705 5488 95.45 5657 5589 | 95.45 |  | 82.9 |  |  | 72 |
| R_Date 5-Beta-587099 | 5705 5488 95.45 5657 5589 | 95.45 |  | 82.9 |  |  | 72.4 |
| R_Date 5-Beta-587094 | 5714 5585 95.45 5658 5593 | 95.45 |  | 110.9 |  |  | 71 |
| R_Date 5-Beta-587096 | 5715 5587 95.45 5659 5595 | 95.45 |  | 119.2 |  |  | 70.2 |
| R_Date 5-Beta-510344 | 5720 5594 95.45 5664 5596 | 95.45 |  | 124.2 |  |  | 67.4 |
| R_Date 5-Beta-587095 | 5728 5596 95.45 5668 5596 | 95.45 |  | 118.8 |  |  | 66 |
| R_Date 5-Beta-587097 | 5844 5600 95.45 5680 5600 | 95.45 |  | 79.6 |  |  | 61.2 |
| R_Date 5-Beta-587098 | 5860 5601 95.45 5681 5601 | 95.45 |  | 67.7 |  |  | 59.6 |
| **Boundary Start Cannibal Event 1** | **5709 …** | **95.45** |  |  |  |  | **70.1** |

| **Boundary End Fumier** |  |  |  | |  | **5530 4931 95.449974** | | | |  | |  | |  | |  | **99,9** |
| --- | --- | --- | --- | --- | --- | --- | --- | --- | --- | --- | --- | --- | --- | --- | --- | --- | --- |
| R_Date 4-Beta-153367 | 5593 | | | 5331 95.45 | | | 5594 | 5334 | 95.45 | |  | | 102 | |  |  | 99.9 |
| R_Date 4-Beta-181086 | 5882 | | | 5596 95.45 | | | 5881 | 5596 | 95.45 | |  | | 99.8 | |  |  | 99.9 |
| R_Date 4-Beta-510345 | 5844 | | | 5600 95.45 | | | 5845 | 5599 | 95.45 | |  | | 99.6 | |  |  | 99.9 |
| R_Date 4-Beta-220912 | 5921 | | | 5739 95.45 | | | 5921 | 5739 | 95.45 | |  | | 99.8 | |  |  | 99.9 |
| R_Date 4-Beta-181087 | 6280 | | | 6000 95.45 | | | 6280 | 6001 | 95.45 | |  | | 99.6 | |  |  | 99.9 |
| R_Date 4-Beta-208131 | 6391 | | | 6192 95.45 | | | 6391 | 6192 | 95.45 | |  | | 99.4 | |  |  | 99.9 |
| R_Date 4-Beta-220913 | 6392 | | | 6198 95.45 | | | 6392 | 6198 | 95.45 | |  | | 99.2 | |  |  | 99.9 |
| R_Date 4-Beta-510342 | 6480 | | | 6305 95.45 | | | 6450 | 6305 | 95.45 | |  | | 99.2 | |  |  | 100 |
| R_Date 4-Beta-510341 | 6491 | | | 6316 95.45 | | | 6491 | 6316 | 95.45 | |  | | 99.6 | |  |  | 99.9 |
| R_Date 4-Beta-181088 | 6657 | | | 6316 95.45 | | | 6657 | 6316 | 95.45 | |  | | 99.9 | |  |  | 99.9 |
| R_Date 4-Beta-510343 | 6787 | | | 6652 95.45 | | | 6787 | 6653 | 95.45 | |  | | 99.8 | |  |  | 99.9 |
| R_Date 4-Beta-197384 | 7158 | | | 6801 95.45 | | | 7158 | 6801 | 95.45 | |  | | 99.8 | |  |  | 99.8 |
| R_Date 4-Beta-220914 | 7159 | | | 6884 95.45 | | | 7159 | 6884 | 95.45 | |  | | 99.7 | |  |  | 99.9 |
| R_Date 4-Beta-208132 | 7158 | | | 6893 95.45 | | | 7158 | 6894 | 95.45 | |  | | 99.7 | |  |  | 99.9 |
| R_Date 4-Beta-182040 | 7164 | | | 6884 95.45 | | | 7164 | 6883 | 95.45 | |  | | 99.9 | |  |  | 99.9 |
| R_Date 4-Beta-208133 | 7164 | | | 6909 95.45 | | | 7164 | 6937 | 95.45 | |  | | 99.9 | |  |  | 99.9 |
| R_Date 4-Beta-208134 | 7420 | | | 7079 95.45 | | | 7420 | 7079 | 95.45 | |  | | 99.8 | |  |  | 99.9 |
| R_Date 4-Beta-197385 | 7423 | | | 7172 95.45 | | | 7423 | 7172 | 95.45 | |  | | 99.8 | |  |  | 99.9 |

*This table presents calibrated radiocarbon dating results, including the range of dates from the model at the 95.4% confidence interval for the different archaeological phases and events. Each row provides the calibrated date ranges for specific samples or events, with 'Boundary' rows indicating the start or end of a phase and 'Interval' rows representing the temporal span of a particular event. The data illustrate the chronological framework for individual and collective burials, as well as episodes indicative of cannibalistic practices, through the Neolithic to the Bronze Age periods.*

*Table S3. Total and frequency of remains with human induced modifications.*

|  |  | *Homo*  *sapiens* (NISP) | *H.*  *sapiens* (NISP*) | Cut Marks | Percussion marks | Peeling | Total  butchering  marks | Burned | Pot polishing | Human  tooth marks |
| --- | --- | --- | --- | --- | --- | --- | --- | --- | --- | --- |
| TEST PIT | MIR1 | 2 | 2 | 0 | 0 | 0 | 0 | 0 | 0 | 0 |
|  | MIR2 | 7 | 5 | 2  (40%) | 0 | 3 (60%) | 3 (60%) | 0 | 4  (80%) | 4 (80%) |
|  | MIR3A | 2 | 2 | 1  (50%) | 0 | 0 | 1 (50%) | 0 | 1  (50%) | 0 |
|  | MIR4 | 157 | 148 | 67  (45.3%) | 29 (19.6%) | 32  (21.6%) | 104  (70.3%) | 3 (2%) | 16  (10.8%) | 75  (50.7%) |
| Sector 100 | MIR101 | 32 | 32 | 2  (6.3%) | 3  (12.5%) | 1  (3.1%) | 4 (12.5%) | 3 (9.4%) | 12  (37.5%) | 5 (15.6%) |
|  | MIR102 | 85 | 83 | 13  (15.3%) | 9 (10.6%) | 5 (5.9%) | 18  (21.2%) | 7 (8.2%) | 55  (64.7%) | 15  (17.6%) |
|  | MIR103 | 5 | 5 | 0 | 0 | 0 | 0 | 0 | 1 (20%) | 1 (20%) |
|  | MIR104 | 6 | 6 | 0 | 0 | 0 | 0 | 0 | 0 | 0 |
|  | MIR105 | 187 | 183 | 12  (6.6%) | 11  (6%) | 3 (1.6%) | 18  (9.8%) | 13  (7.1%) | 46  (25.1%) | 16  (8.7%) |
|  | MIR106 | 152 | 145 | 0 | 0 | 0 | 0 | 1  (0.7%) | 0 | 0 |
|  | MIR107 | 6 | 6 | 0 | 0 | 0 | 0 | 0 | 0 | 0 |
|  | MIR108 | 9 | 9 | 0 | 0 | 0 | 0 | 0 | 0 | 0 |
| Sector 200 | MIR201 | 1814 | 1736 | 8  (0.5%) | 10  (0.6%) | (0.3%) | 16  (0.9%) | 9  (0.5%) | 45  (2.6%) | 11  (0.6%) |
|  | MIR202 | 1539 | 1508 | 57  (3.8%) | 51  (3.4%) | 21  (1.4%) | 100  (6.6%) | 88  (5.8%) | 269  (17.8%) | 73  (4.8%) |
|  | MIR203 | 726 | 692 | 7(1%) | 10  (1.4%) | 0 | 12  (1.7%) | 10  (1.4%) | 25  (3.6%) | 2 (0.3%) |
|  | MIR204 | 184 | 182 | 29  (15.9%) | 47  (25.8%) | 5 (2.7%) | 56  (30.8%) | 37  (20.3%) | 105  (57.7%) | 30  (16.5%) |
|  | MIR205 | 98 | 91 | 5 (5.5%) | 6 (6.6%) | 4 (4.4%) | 12  (13.12%) | 20  (22%) | 44  (48.4%) | 8 (8.8%) |
|  | MIR206 | 45 | 44 | 0 | 2 (4.5%) | 0 | 2 (4.5%) | 1 (2.3%) | 3 (6.8%) | 1 (2.3%) |
|  | Total | 5056 | 4879 | 203  (4.2%) | 178  (3.6%) | 76  (1.5%) | 346  (7.1%) | 189  (3.9%) | 627  (12.8%) | 162  (3.3%) |

*The table show the frequency of cut marks, percussion marks, peeling (butchering marks), burning, pot polishing, and human tooth marks on the human bones in the different stratigraphic units from El Mirador cave. *NISP without isolated teeth, The isolated teeth have been excluded from the frequencies of modifications calculation.*

*Table S4. Remains with anthropogenic modifications distributed by anatomical elements from the S100 and S200.*

|  | Cut marks | Percussion Marks | Peeling | Burned Bones | Pot polishing |
| --- | --- | --- | --- | --- | --- |
| Skull | 6 | 13 | 1 | 14 | 14 |
| Mandible | 2 | 5 | 2 | 3 | 5 |
| Clavicle | 3 | 0 | 1 | 4 | 4 |
| Vertebra | 2 | 0 | 3 | 2 | 4 |
| Rib | 19 | 2 | 3 | 12 | 28 |
| Coxa | 1 | 1 | 0 | 1 | 1 |
| Scapula | 4 | 1 | 1 | 3 | 4 |
| Humerus | 18 | 14 | 0 | 9 | 21 |
| Radius | 4 | 3 | 1 | 3 | 5 |
| Ulna | 8 | 10 | 2 | 4 | 15 |
| Carpal | 0 | 0 | 0 | 0 | 0 |
| Femur | 27 | 40 | 0 | 20 | 34 |
| Patella | 0 | 0 | 0 | 0 | 0 |
| Tibia | 22 | 41 | 0 | 16 | 41 |
| Fibula | 9 | 15 | 3 | 2 | 18 |
| Tarsal | 0 | 0 | 0 | 5 | 0 |
| Metapodial | 4 | 3 | 2 | 3 | 7 |
| Phalange | 2 | 0 | 4 | 2 | 6 |
| Total | 132 | 148 | 23 | 103 | 207 |

*Table S5. Specimens directly dated*

| **Reference** | **Element** | **Description** | **Modifications** |
| --- | --- | --- | --- |
| ATA10-MIR101-SC-48 | Tibia left | Complete shaft | Percussion marks |
| ATA13-MIR105-V14-102 | Femur right | Midshaft | Cut and Percussion marks |
| ATA09-MIR201-SC-5 | Humerus right | Midshaft and distal epiphysis | Cut marks |
| ATA12-MIR203-S37-13 | Femur right | Proximal and midshaft | Cut and Percussion marks |
| ATA15-MIR204-R36-2 | Tibia right | Midshaft | Cut and Percussion marks |
| ATA15-MIR204-S35-18 | Femur left | Midshaft | Cut marks |
| ATA17-MIR205-Q36-1 | Humerus right | Distal shaft | Cut marks |
| ATA18-MIR206-Q38-10 | Humerus right | Midshaft young | Percussion marks |

*Table S6. NISP, MNE and MNI of the remains with human induced modifications from sectors 100 and 200.*

|  | NISP | MNE | MNI |
| --- | --- | --- | --- |
| Skull | 17 | 4 | 4 |
| Mandible | 5 | 4 | 4 |
| Clavicle | 4 | 4 | 3 |
| Vertebra | 4 | 4 | 1 |
| Rib | 32 | 18 | 1 |
| Coxa | 1 | 2 | 1 |
| Scapula | 6 | 5 | 3 |
| Humerus | 25 | 7 | 4 |
| Radius | 6 | 5 | 3 |
| Ulna | 15 | 9 | 6 |
| Femur | 46 | 14 | 9 |
| Tibia | 44 | 7 | 4 |
| Fibula | 20 | 8 | 6 |
| Metapodial | 8 | 7 | 1 |
| Phalange | 6 | 6 | 1 |
| Total | 239 | 104 | 9 |

*Table S7. Synthesis of the minimal number of individuals and the death age.*

| Individual 1 | Skull | 2011-MIR105-W15-10 and 2011-MIR105-  V15-38 | < 5-7 years old |
| --- | --- | --- | --- |
| Individual 2 | Skull | 2015-MIR105-T14-86 | < 5-7 years old |
| Individual 3 | Mandible | 2016-MIR202-N37-58 | 6-10 years old |
| Individual 4 | Mandible | 2016-MIR102-S13-20 | 15-17 years old |
| Individual 5 | Mandible | 2010-MIR202-T35-11 | 12-15 years old |
| Individual 6 | Maxilla | 2016-MIR102-T12-21 | 20-25 years old |
| Individual 7 | Mandible | 2016-MIR102-T12-6 | >50 years old |
| Individual 8 | Femur | - | Adult |
| Individual 9 | Femur | - | Adult |
| Individual 10? | Femur | - | Indet |
| Individual 11? | Femur | - | Indet |

*Table S8. 87Sr/86Sr results for the tested samples***.**

| Lab ID | Reference | Bone  identification | 87Sr/86Sr  Raw | ±95%CI | 87Sr/86Sr Adjusted* | ±95%CI |
| --- | --- | --- | --- | --- | --- | --- |
| IS-1230 | ATA2016-MIR204-O38-6 | Femur | 0,709808 | 0,000017 | 0,709814 | 0,000017 |
| IS-1231 | ATA2015-MIR204-Q37-  11 | Femur | 0,709162 | 0,000096 | 0,709169 | 0,000096 |
| IS-1232 | ATA2015-MIR204-P37-39 | Femur | 0,710581 | 0,000138 | 0,710587 | 0,000138 |
| IS-1233 | AA2013-MIR202-P39-9 | Femur | 0,709721 | 0,000019 | 0,709727 | 0,000019 |
| IS -1234 | ATA2012-MIR203-S37-13 | Femur | 0,709759 | 0,000072 | 0,709765 | 0,000072 |

**Adjusted relative to the accepted value of 0.710248±0.000003 (MacArthur et al., 2001) for SRM 987.*

# References

1. [Vergès, J. M. *et al.* El Mirador Cave: Biogeographical Setting and Site Description.](http://paperpile.com/b/x4U1di/qPhR)

[in *Prehistoric Herders and Farmers: A Transdisciplinary Overview to the Archeological Record from El Mirador Cave* (eds. Allué, E., Martín, P. & Vergès, J. M.) 13–34 (Springer International Publishing, Cham, 2022).](http://paperpile.com/b/x4U1di/qPhR)

1. [Vergès, J. M. *et al.* El Mirador cave (Sierra de Atapuerca, Burgos, Spain): A whole perspective. *Quat. Int.* **414**, 236–243 (2016).](http://paperpile.com/b/x4U1di/829U)
2. [Allué, E., Martín, P. & Vergès, J. M. *Prehistoric Herders and Farmers: A Transdisciplinary Overview to the Archeological Record from El Mirador Cave*. (Springer Nature, 2022).](http://paperpile.com/b/x4U1di/HcuX)
3. [Burguet-Coca, A. *et al.* The Fumier Sequences of El Mirador: An Approach to Fire as a Sociocultural Practice and Taphonomic Agent. in *Prehistoric Herders and FarmersA Transdisciplinary Overview to the Archeological Record from El Mirador Cave* (ed. Allué, E., Martín, P., Vergès, J.M.) 89–110 (Springer Cham, 2022).](http://paperpile.com/b/x4U1di/9zlJ)
4. [Angelucci, D. E., Boschian, G., Fontanals, M., Pedrotti, A. & Vergès, J. M. Shepherds and karst: the use of caves and rock-shelters in the Mediterranean region during the Neolithic. *World Archaeol.* **41**, 191–214 (2009).](http://paperpile.com/b/x4U1di/DbUU)
5. [Carrancho, Á., Herrejón Lagunilla, Á. & Vergès, J. M. Three archaeomagnetic applications of archaeological interest to the study of burnt anthropogenic cave sediments. *Quaternary International* **414**, 244–257 (2016).](http://paperpile.com/b/x4U1di/V5N7)
6. [Herrejón-Lagunilla, Á., Carrancho, Á. & Villalaín, J. J. On the Suitability of](http://paperpile.com/b/x4U1di/IFDA)

[Prehistoric Anthropogenic Burnt Sediments (Fumiers) for Archeomagnetic Studies at El Mirador Cave (Burgos, Spain). in *Prehistoric Herders and Farmers: A*](http://paperpile.com/b/x4U1di/IFDA)

[*Transdisciplinary Overview to the Archeological Record from El Mirador Cave* (eds. Allué, E., Martín, P. & Vergès, J. M.) 111–128 (Springer International Publishing, Cham, 2022).](http://paperpile.com/b/x4U1di/IFDA)

1. [Schaefer, M. C., Scheuer, L. & Black, S. *Juvenile Osteology: A Laboratory and Field Manual*. (Academic Press, 2014).](http://paperpile.com/b/x4U1di/ah8y)
2. [Saladié, P. *et al.* Experimental Butchering of a Chimpanzee Carcass for Archaeological Purposes. *PLoS One* **10**, e0121208 (2015).](http://paperpile.com/b/x4U1di/d6KP)
3. [Boulestin, B. & Coupey, A.-S. *Cannibalism in the Linear Pottery Culture: The Human Remains from Herxheim*. (Archaeopress Publishing Limited, 2015).](http://paperpile.com/b/x4U1di/9V2c)
4. [Pickering, T. R. *et al.* Taphonomy of ungulate ribs and the consumption of meat and bone by 1.2-million-year-old hominins at Olduvai Gorge, Tanzania. *J. Archaeol. Sci.* **40**, 1295–1309 (2013).](http://paperpile.com/b/x4U1di/zGNs)
5. [Saladié, P., Rodríguez-Hidalgo, A., Díez, C., Martín-Rodríguez, P. & Carbonell, E.](http://paperpile.com/b/x4U1di/tX5v)

[Range of bone modifications by human chewing. *J. Archaeol. Sci.* **40**, 380–397 (2013).](http://paperpile.com/b/x4U1di/tX5v)

1. [Fernández-Jalvo, Y. & Andrews, P. When humans chew bones. *J. Hum. Evol.* **60**, 117–123 (2011).](http://paperpile.com/b/x4U1di/DxZy)
2. [Vergès, J. M. *et al.* La Sierra de Atapuerca durante el Holoceno: datos preliminares sobre las ocupaciones de la Edad del Bronce en la Cueva de El Mirador (Ibeas de Juarros, Burgos). *Trabajos de Prehistoria* **59**, 107–126 (2002).](http://paperpile.com/b/x4U1di/D1Rz)
3. [Bisbal-Chinesta, J. F. *et al.* Elucidating anuran accumulations: massive taphocenosis of tree frog Hyla from the Chalcolithic of El Mirador cave (Sierra de Atapuerca, Spain). *Journal of Archaeological Science: Reports* **30**, 102277 (2020).](http://paperpile.com/b/x4U1di/OSnc)
